# Contribution of proteomics and metabolomics data to understanding of health benefits of tea

**DOI:** 10.1016/j.chmed.2025.11.009

**Published:** 2025-11-20

**Authors:** Danicke Willemse, Mariam Rado, Mariska Lilly

**Affiliations:** Applied Microbial and Health Biotechnology Institute, Cape Peninsula University of Technology, Bellville 7535, South Africa

**Keywords:** *Aspalathus linearis* (Burm.f.) R.Dahlgren, *Camellia sinensis* (L.) Kuntze, *Cyclopia* species, metabolomics, proteomics, tea

## Abstract

Tea is the second most widely consumed non-alcoholic beverage globally. While most teas originate from *Camellia sinensis* (L.) Kuntze plants, rooibos and honeybush teas are produced from *Aspalathus linearis* (Burm.f.) R.Dahlgren and *Cyclopia* species tea plants. Interest in tea and tea-derived components, has increased due to their well-known health benefits. The mechanisms of these health benefits are however poorly understood. Proteomics and metabolomics provide valuable tools to assess the mechanisms of the therapeutic effects of tea in disease treatment. This review summarizes the role played by proteomic and metabolomic studies in investigating the health benefits of *C. sinensis*, *A. linearis,* and *Cyclopia* spp*.* teas. Surprisingly, no proteomic and metabolomic studies investigating the health benefits of *A. linearis* and *Cyclopia* spp*.* teas and/or their components were identified in a literature search. However, 25 studies using proteomics and 16 studies using metabolomics to investigate the health benefits of *C. sinensis* teas and/or their components were identified in a literature search. Proteomics and metabolomics have been valuable tools for investigating the health benefits of *C. sinensis* teas and tea components, and will likely also prove valuable for investigating the effects of *A. linearis* and *Cyclopia* spp. teas on human health.

## 1 Introduction

**Fig. 1:**
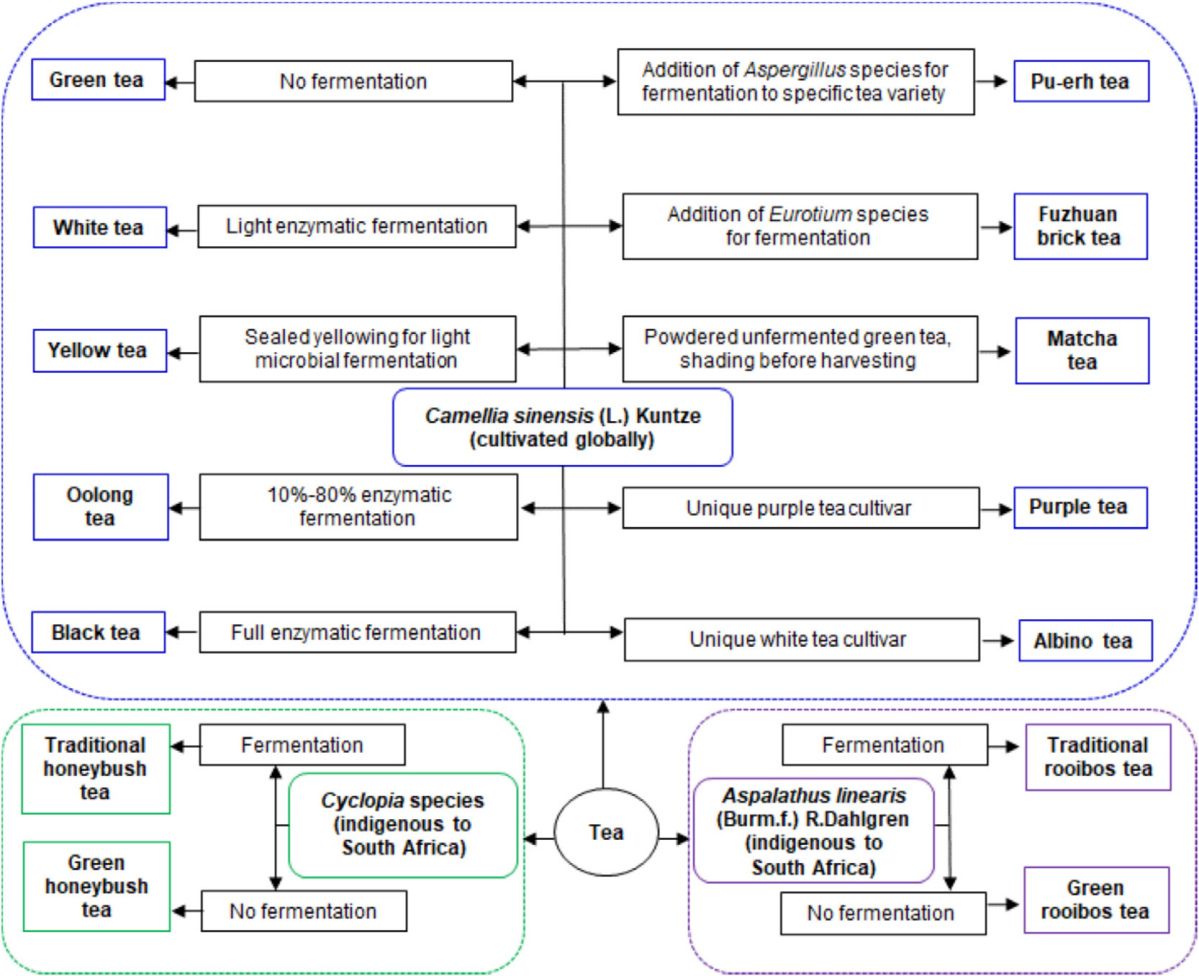
Various teas produced from tea plants.

Tea is the second most widely consumed non-alcoholic beverage globally, after water. A variety of teas are derived from the *Camellia sinensis* (L.) Kuntze plant which is cultivated globally (Wong, Sirisena, & Ng, 2022). Rooibos and honeybush teas are produced from indigenous South African plants, *Aspalathus linearis* (Burm.f.) R.Dahlgren and *Cyclopia* species, respectively (Joubert et al., 2019) (Fig. 1). Teas produced from these three tea plants are abundant in bioactive compounds, specifically phytochemicals, which exhibit biological activities and influence metabolic processes. The health benefits of teas have been extensively documented. In recent decades, there has been growing interest in exploring tea as an alternative or complementary treatment for various human diseases. Despite the identification of many health benefits, the mechanisms responsible for these effects remain largely unknown (Afrifa et al., 2023, Joubert et al., 2019, Kumari and Kumar, 2024).

Proteomics and metabolomics are powerful tools for investigating the molecular mechanisms that underlie the health benefits of natural products (Hasanpour et al., 2020, Khan et al., 2022, Lao et al., 2014, Oyenihi et al., 2021, Shi et al., 2016). This review seeks to synthesize findings from proteomic and metabolomic studies and highlight how these approaches have contributed to understanding the health-promoting effects of *C. sinensis*, *A. linearis*, and *Cyclopia* spp*.* teas and their bioactive compounds.

## 2 Classification of teas

### 2.1 C. sinensis teas

*C. sinensis,* from the Theaceae family, is cultivated globally for tea production. Teas are classified based on demographics, processing, and fermentation level (Wong, Sirisena, & Ng, 2022) and named according to its region of origin (Fig. 1).

Green tea: Unfermented tea produced by steaming or pan-firing tea leaves to prevent enzymatic fermentation and preserve high catechin levels (Wong, Sirisena, & Ng, 2022).

White tea: Minimally processed tea, often produced from young buds of tea, with a unique composition shaped by prolonged withering process during which light enzymatic fermentation occurs (Wong, Sirisena, & Ng, 2022).

Yellow tea: Like green tea but uses younger leaves and a longer heating at lower-temperatures for pan-firing tea leaves. Its unique composition is shaped by sealed yellowing during which microbial fermentation, by microbes occurring naturally on the plant, occurs (Xu et al., 2018).

Oolong tea: Partial enzymatic fermentation (10%−80%) is done to produce six distinct varieties (Wong, Sirisena, & Ng, 2022).

Black tea: Rolling and maceration of tea leaves are done to ensure full enzymatic fermentation and, depending on rolling technique, various types of black tea can be produced (Wong, Sirisena, & Ng, 2022).

Pu-erh tea: It is made from a unique tea variant, *C. sinensis* var*. assamica*, which has larger leaves that are pan-fried, sun-dried, and fermented using *Aspergillus* species (Wong, Sirisena, & Ng, 2022).

Fuzhuan brick tea: A dark tea produced by fermentation with *Eurotium* fungi for 2−3 weeks producing a distinct metabolomic composition (Xu, Hu, Wang, Wan, & Bao, 2015).

Matcha tea: A finely powdered Japanese green tea made from shaded young shoots, enhancing its bioactive compounds. Including whole leaves, unlike standard green tea (Koláčková et al., 2020).

Purple tea: Produced similarly to green tea but from a unique purple tea variant, *C. sinensis* var. *assamica* cv. *Zijuan*, leading to a unique composition (Li et al., 2021b).

Albino tea: Produced from mutated *C. sinensis* plants lacking chlorophyll, leading to altered metabolism. White tea and albino-induced yellow tea can also be made from these cultivars (Li et al., 2018).

### 2.2 A. linearis teas

*A. linearis*, from the Fabaceae family and Fynbos Biome, is the primary species used for rooibos tea production, though other *Aspalathus* species were historically used (Van Wyk & Gorelik, 2017). Unlike *C. sinensis* teas, the entire plant, except flowers, is used to produce two types of rooibos teas (Fig. 1).

Traditional rooibos tea: *A. linearis* plant is shredded, bruised, and mixed with water to ferment overnight, the tea is sun-dried and lumps are removed by sieving (Joubert and Schulz, 2006).

Green rooibos tea: *A. linearis* plant is either dried at low temperatures with controlled humidity or exposed to steam to inactivate enzymes and microbes responsible for fermentation. The plant is then shredded, sun-dried, and sieved (Joubert and Schulz, 2006).

### 2.3 Cyclopia spp. Teas

*Cyclopia* species, endemic to South Africa’s Fynbos Biome, belong to the Fabaceae family. Many of the 23 species have historically been used for honeybush tea production (Joubert et al., 2019, Van Wyk and Gorelik, 2017).

Traditional honeybush tea: Chopped *Cyclopia* stems and leaves are wetted and fermented, with oven heating used to regulate the process. Once the desired properties are developed, fermentation is stopped by sun drying, followed by sifting to remove clumps (Joubert et al., 2019).

Green honeybush tea: Chopped *Cyclopia* stems and leaves undergo brief high-temperature steaming to prevent fermentation. The tea is then dried using vacuum or sun drying and sieved to remove clumps (Joubert et al., 2019).

## 3 Bioactive compounds in teas

### 3.1 Bioactive compounds in C. sinensis teas

Differential processing of the *C. sinensis* plants results in variations in their biochemical composition. In green tea, the primary bioactive compounds are catechins, a class of natural polyphenolic phytochemicals. These include epigallocatechin gallate (EGCG), epigallocatechin (EGC), epicatechin (EC), gallocatechin gallate (GCG), epicatechin gallate (ECG), gallocatechin (GC), and catechin (Dai et al., 2017, Kim et al., 2016, Turkmen et al., 2009, Wong et al., 2022). During the fermentation and further processing of green tea, catechins are transformed into derivatives such as theaflavins, thearubigins, and theabrownins. The major theaflavins include theaflavin-3-gallate, theaflavin-3′-gallate, and theaflavin-3,3′-digallate. Thearubigins are formed through the oxidation of theaflavins or the polymerization of proanthocyanidins, while procyanidins and theasinesins are dimeric catechins (Dai et al., 2017, Tan et al., 2016). Theabrownins are produced through further oxidation of theaflavins and thearubigins (Turkmen et al., 2009, Zhao et al., 2022). In addition to catechins and their derivatives, other bioactive compounds found in *C. sinensis* teas include methylated catechins (Dai et al., 2017), methylxanthines such as caffeine, theobromine, and theophylline (Abdullah et al., 2024, Wong et al., 2022), theacrine (a purine alkaloid) and *L*-theanine, a nonproteinogenic amino acid, among others (Fang et al., 2017, Wong et al., 2022).

### 3.2 Bioactive compounds in A. linearis teas

Many of the bioactive compounds present in rooibos tea are unique compared to those found in *C. sinensis* teas. Rooibos teas are high in polyphenols and flavonoids, including aspalathin, nothofagin, *iso*-orientin, orientin, vitexin, isovitexin, quercetin, isoquercetin, rutin, luteolin, hyperoside and eriodictyol-*C*-glucosides, among others (Malongane et al., 2018, Masike, 2021, Stander et al., 2017, Tobin, 2018). Fermentation causes oxidation and therefore changes the composition of rooibos tea significantly, leading to lower aspalathin and nothofagin levels and higher eriodictyol-*C*-glucosides (De Beer et al., 2019, Tobin, 2018).

### 3.3 Bioactive compounds in Cyclopia spp. teas

The metabolic composition of *Cyclopia* spp. vary (Karsen, Lötze, Valentine, & Hoffman, 2022), and is distinct from rooibos and *C. sinensis* teas (Malongane et al., 2018, Masike, 2021). Various polyphenols, flavonoids, xanthones, benzophenones and dihydrochalcones, including mangiferin, isomangiferin, hesperidin, orientin, vicenin-2, quercetin, luteolin and vitexin (among others), are found in *Cyclopia* species, with mangiferin being the major polyphenol (Joubert et al., 2019, Karsen et al., 2022, Malongane et al., 2018, Masike, 2021). Fermentation causes oxidation of polyphenols, thereby decreasing their levels in fermented honeybush (Joubert et al., 2019).

## 4 Health benefits of teas

### 4.1 Health benefits of C. sinensis teas

The diverse health benefits of *C. sinensis* teas, tea extracts, and bioactive compounds are well-known and have been thoroughly reviewed in recent literature (Aloo et al., 2024, Chaudhary et al., 2023, Das et al., 2022, El Oirdi, 2024, Kumari and Kumar, 2024, Mandal and Seth, 2023, Teixeira and Sousa, 2021). In summary, *C. sinensis* teas possess antioxidant and anti-inflammatory properties, making them effective in treating inflammatory diseases. Additionally, they exhibit anticancer and antitumour properties, with evidence suggesting their potential in preventing malignancies and acting against various cancers. Tea also shows protective effects on eye health, particularly in its anti-glaucoma activity, and has neuroprotective potential, with promising findings in Alzheimer’s disease research. Furthermore, tea helps in managing metabolic disorders by lowering blood glucose levels and providing protection against hyperlipidaemia and atherosclerosis. These actions contribute to its anti-obesity and anti-diabetic properties, along with its ability to reduce hypertension and enhance cardiovascular health. In terms of digestive health, tea can protect against digestive disorders and exhibit prebiotic activity. While beneficial to gut microbiota, tea also displays antibacterial, antifungal, and antiviral activities against pathogenic microbes, thereby aiding in wound protection and infection prevention. Its inclusion in cosmetic products is also common due to its numerous skin health benefits (Aloo et al., 2024, Chaudhary et al., 2021, Das et al., 2022, El Oirdi, 2024, Kumari and Kumar, 2024, Mandal and Seth, 2023, Teixeira and Sousa, 2021).

### 4.2 Health benefits of A. linearis teas

Various review papers summarizing the role of rooibos teas in human health have been published recently (Abdul and Marnewick, 2023, Afrifa et al., 2023, Chaudhary et al., 2021, Joubert et al., 2023, Maarman and Lecour, 2022, Pretorius and Smith, 2024, Pringle et al., 2018). In summary, rooibos tea has anti-oxidant activity therefore alleviating oxidative stress as well as anti-inflammatory properties allowing its use to treat inflammatory diseases and stress management. These properties also contribute to the anticancer, antimutagenic, antitumour and antiapoptotic abilities of rooibos teas. Many studies have focused on the benefits of rooibos tea to metabolic disorders and highlight the ability of rooibos to lower blood glucose levels, improve lipid profiles and help prevent obesity and diabetes development. Beneficial effects on cardiac health and diseases, such as pulmonary arterial hypertension as well as antithrombotic properties, have also been observed. Rooibos tea is also beneficial to bone health as well as intestinal health, having antispasmodic properties as well as prebiotic activity. Dermatological products containing rooibos have also become very common, because of the antiwrinkle, antiaging and ultraviolet radiation B (UVB) protective effects. It is also able to speed up wound recovery and has antibacterial activity (Abdul and Marnewick, 2023, Afrifa et al., 2023, Chaudhary et al., 2021, Joubert et al., 2023, Keet et al., 2024, Maarman and Lecour, 2022, Pretorius and Smith, 2024, Pringle et al., 2018). These reviews therefore highlight the positive effects of rooibos tea on the whole body.

### 4.3 Health benefits of Cyclopia spp. teas

*Cyclopia* spp. teas have numerous health benefits which have been reviewed recently (Joubert et al., 2019, Windvogel, 2019). Honeybush tea has anti-inflammatory, anti-oxidant, anti-proliferation and pro-apoptotic effects. These attributes contribute to honeybush tea having anticancer and antitumour effects. Since it contains phytoestrogens, it can regulate oestrogen levels. Honeybush tea also helps against metabolic syndrome, due to its ability to lower blood glucose levels, increase glucose absorption, improve glucose tolerance and lower blood lipid levels. It therefore is supportive to preventing diabetes and obesity. Additionally, it has been shown to improve cardiovascular health. It is also common to find honeybush extracts in dermatological products as it has been shown to improve skin wrinkles, elasticity, and hydration, and have antiaging and antiwrinkle properties as well as providing protection from ultraviolet (UV) damage. Honeybush teas also have antimicrobial activity against some pathogenic microbes (Joubert et al., 2019, Keet et al., 2024, Windvogel, 2019). Honeybush tea has also been shown to prevent oxidative damage and mitochondrial dysfunction which are related with aging as well as neurodegenerative and mental disorders. It is therefore suggested to help against neurodegenerative diseases (Agapouda et al., 2020). Honeybush tea also decreases drug induced liver injury through preventing oxidative stress, thereby showing hepatoprotectant properties (Reddy et al., 2023). It is therefore clear that honeybush tea has positive effect on the overall health of the whole body.

While the health benefits of teas are widely recognized, the precise molecular mechanisms underlying these effects remain largely unclear. Gaining a deeper understanding of these mechanisms is essential for optimizing the use of teas as potential alternative or complementary treatments for various human diseases. Proteomics and metabolomics offer powerful approaches to identify the molecular pathways influenced by tea and clarify the mechanisms of action of its bioactive compounds. This review therefore seeks to summarize the way in which proteomics and metabolomics have supported our understanding of the health benefits of teas.

## 5 Role of proteomics studies in determining health benefits of teas

**Fig. 2:**
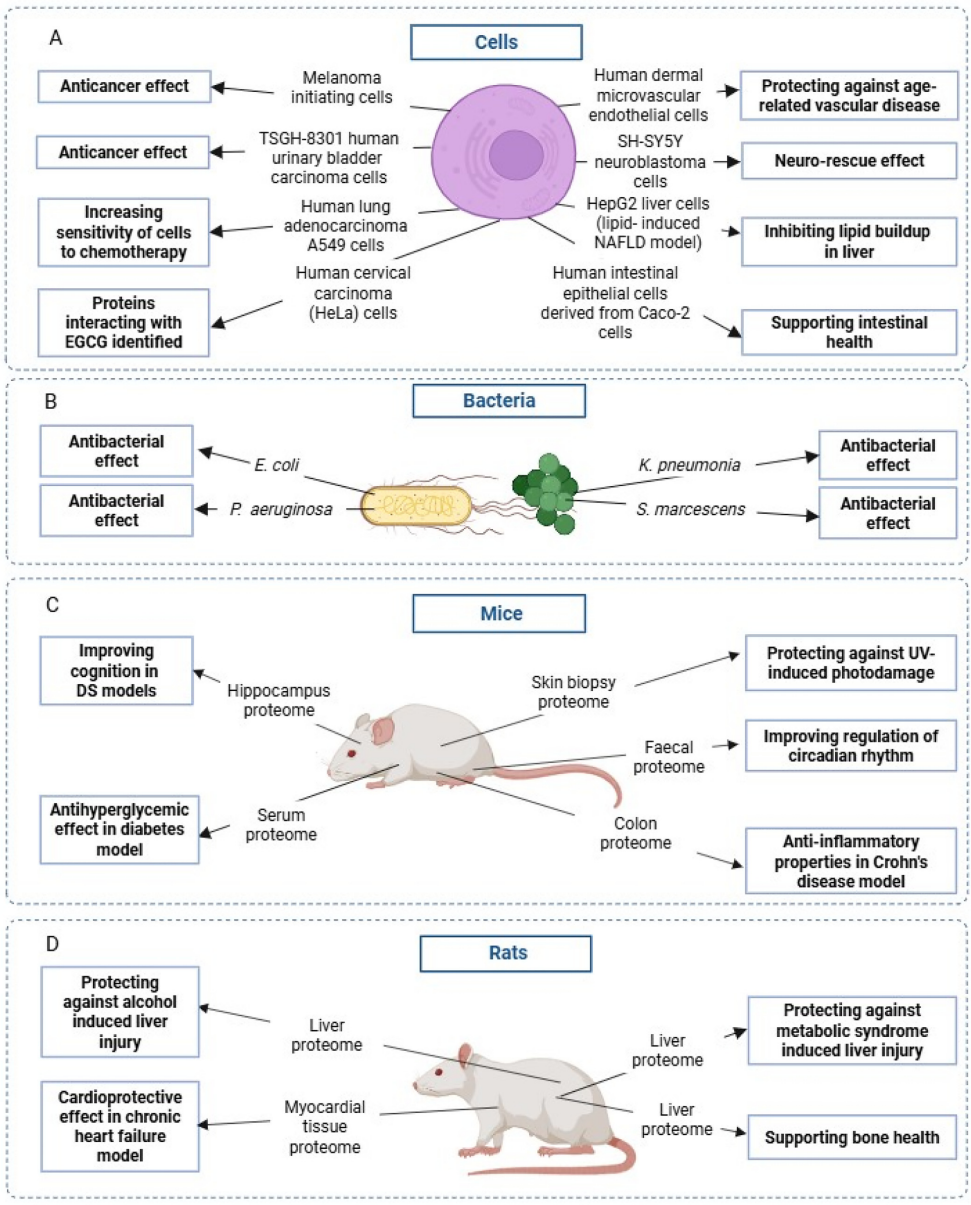
Various models used for investigating health benefits of *C. sinensis* teas by proteomic analyses including (A) cells, (B) bacteria, (C) mice and (D) rats. This image was created in Biorender.com.

A literature search identified 25 studies, which investigated the health benefits of *C. sinensis* teas and tea components using proteomics. No proteomics studies investigating the health benefits of *A. linearis* and *Cyclopia* spp*.* were identified. The proteomics strategies used in *C. sinensis* studies, organized in terms of separation techniques and mass spectrometry (MS) strategy used, are summarized in Table 1. The various models used for investigating the health benefits of *C. sinensis* teas by proteomic analyses are summarized in Fig. 2.

**Table 1:** Proteomic studies aimed at understanding health benefits of *C. sinensis* teas.

	Teas/compounds*	Study objectives	Models	Tissues/sub-cellular fractions	Proteomics strategies*		Key findings	References
					Separation	Identification		
P1	Green tea	Antihyperglycemic properties	Diabetic mouse model	Serum proteome	Protein Chip array CM10	SELDI-TOF MS	Green tea decreased unidentifiable 4 211 protein level thought to be related to haemoglobin	Tsuneki et al., 2004
P2	EGCG	Synergistic effect of EGCG and imipenem in imipenem-resistant *K. pneumonia*	Imipenem-resistant *K. pneumonia* clinical isolates	Outer membrane proteins	2DE	MALDI-TOF MS	EGCG increased stress response and detoxification and decreased energy metabolism, protein synthesis and DNA metabolism proteins	Cho, Oh, & Oh, 2011
P3	GTP	Mechanisms protecting against bone loss due to depleted oestrogen	Ovariectomized rats	Liver proteome	2DE	MALDI-TOF MS	GTP had antioxidant activity and decreased catechol-*O*-methyltransferase supporting bone health	Shao, Chen, Lu, Shen, & Gao, 2011
P4	EGCG	EGCG inhibits lipid buildup in the liver	HepG2 liver cells with induced lipid accumulation mimicking NAFLD model	−	2DE	MALDI-TOF MS	EGCG suppressed hepatic gluconeogenesis, improved fat oxidation, and modified redox balance	Liu et al., 2013
P5	TP	Antibacterial effects	*E. coli* (ATCC 25922)	−	2DE	MALDI-TOF/TOF MS	Cellular defence proteins were more abundant, carbon and energy metabolism and amino acid biosynthesis were less abundant in the presence of tea polyphenols	Cho, Schiller, Kahng, & Oh, 2007
P6	TP	Role of cell membrane damage in the antibacterial effect	*P. aeruginosa*	−	2DE	MALDI-TOF/TOF MS	Tea likely caused metabolic disorder in combination disruption of the membrane integrity, inhibiting *P. aeruginosa*	Yi, Zhu, Fu, & Li, 2010
P7	GTP	Antibacterial effect	*S. marcescens*	Soluble membrane proteins	2DE	MALDI-TOF/TOF MS	GTP disrupted the cell membrane and caused metabolic disorder thereby inhibiting bacterial growth	Yi et al., 2014
P8	EGCG	Mechanism of EGCG in preventing age-related vascular diseases	HDMEC cells	−	2DE	MALDI-TOFu MS	EGCG induced proteins related to cytoskeleton formation	Lee, Chung, Bang, & Lee, 2006
P9	GTE	Molecular targets involved in prevention of cell motility and therefore inhibiting lung cancer malignant development	Human lung adenocarcinoma A549 cells	−	2DE	Nano-ESI-QTOF MS	GTEs decreased cell motility, in part, by increasing cell stiffness through increased lamin protein production	Lu et al., 2009, Zamanian-Azodi et al., 2021
P10	EGCG	Mechanism of neurorescue effect	Long-term serum starved SH-SY5Y neuroblastoma cells	−	2DE + RPC	Ion-trap mass spectrometer	Neurorescue effect of EGCG may be associated with its antioxidant and iron chelating effect, thereby preventing oxidative neuronal death	Weinreb et al., 2007, Weinreb et al., 2008
P11	EGCG	Mechanisms involved in EGCG-triggered apoptosis	TSGH-8301 human urinary bladder carcinoma cells	−	2DE + RPC	QTOF-MS	EGCG modulated AKT and HSP27 proteins, activating intrinsic apoptotic pathway	Chen et al., 2011
P12	GTP	Anti-inflammatory efficacy in treating colon inflammation	Mdr1a Crohn’s disease mouse model	Colon tissue proteome	2DE + RPC	Linear Trap Quadrupole MS/MS	Tea altered cellular stress, immune response, and inflammation, cellular assembly, organizational processes, and signalling pathway proteins	Barnett et al., 2013
P13	Fuzhuan brick tea	Effect of tea on metabolic syndrome induced liver changes	Male SD rats with induced metabolic syndrome	Liver proteome	2DE + RPC	High-Capacity-Ion-Trap MS	Fuzhuan tea reduced lipogenesis and glycolysis while enhancing fatty acid oxidation	Liu et al., 2015
P14	EGCG	Identifying novel chemotherapeutic targets of EGCG	Human lung adenocarcinoma A549 cells	−	2DE + RPC	ESI-MS/MS	EGCG supressed HDGF protein enhancing, thereby chemotherapy-induced apoptosis	Flores-Pérez et al., 2016
P15	Theophylline	Anticancer effects	SK-Mel-1 and SK-Mel-2 cells	Post-nuclear cell extract proteome	1DE + RPC	Linear Trap Quadrupole-MS/MS	Theophylline induced dendrite formation and cytoskeleton dynamics and therefore cellular differentiation	Cordella et al., 2019
P16	EGCG	Molecular mechanism of cardioprotective effects	Chronic heart failure model induced in rats	Myocardial tissue proteome	RPC	Hybridquadrupole-Orbitrap MS	EGCG improved cardiac dysfunction in part through improved myocardial energy metabolism	Mou et al., 2022
P17	Oolong tea	Mechanism in regulation of circadian rhythm	C57BL/6J mice colonized with human stool and exposed to darkness to disrupt circadian rhythm	Faecal proteome	RPC	Q Exactive mass spectrometer	Oolong tea influenced carbohydrate and amino acid metabolism, global and overview maps, genetic and environmental information processing leading to adaptation of the microbiome	Guo et al., 2019
P18	EGCG	Effect of EGCG on intestinal exosomes	Human intestinal epithelial cells derived from Caco-2 cells	Exosomes proteome	RPC	Q-Exactive Plus mass spectrometer	EGCG increased wound healing, regulation of bodily fluid levels, skin development’ and negative regulation of blood coagulation proteins	Yano, Suzuki, & Hara, 2023
P19	DBTVS	Anti-alcoholic effect on liver injury	SPF male SD rats given alcohol and DBTVS	Liver proteome and transcriptome	RPC	Orbitrap Exploris 480	The combined proteomic and transcriptomic analyses indicated that DBTVS inhibited several lipid metabolism-related proteins	Gao et al., 2023
P20	GTE	Mechanism of improved cognition	Transgenic mice overexpressing Dyrk1A gene as DS model	Hippocampus proteome and phospho-proteome	Phospho-peptides enriched by TSC, RPC	Linear ion trap, Orbitrap Velos Pro mass spectrometer	Green tea and EE rescued antioxidant activity and phospho-proteins involved in synaptic-neurogenesis enhancing cognition	De Toma, Ortega, Aloy, Sabidó, & Dierssen, 2019
P21	GTE	Mechanism of improved cognition	Ts65Dn, trisomy of chromosome 21 mouse model as DS model	Hippocampus proteome and phospho-proteome	Phospho-peptides enriched by TSC, RPC	Linear ion trap, Orbitrap Velos Pro mass spectrometer	Green tea and EE rescued proteins involved in neurodevelopment, chromatin, immune response, cytoskeleton and GTPase activity and re-establish proper epigenetic state thereby enhancing cognition	De Toma et al., 2020
P22	EGCG	Antibacterial activity	*S. suis*	−	RPC, TMT-labelling	QTOF/MS	EGCG altered proteins involved in cell development and division, components of cell walls and membranes, drug resistance, environmental adaptation, and haemolytic activity. This was correlated with metabolomic studies	Gao et al., 2022a
P23	Theacrine	Mechanism of prevention of UV-induced photodamage in skin	Female ICR mice exposed to UV	Skin biopsy proteome	1DE + RPC TMT-labelling	MS/MS	Theacrine reduced UVB-induced epidermal damage by altering the signalling cascade associated with apoptosis	He et al., 2023
P24	Biotin- tagged EGCG	Identify proteins interacting with EGCG	HeLa cells	−	Biotin-tagged EGCG, Isotope-labelling, RPC	QTOF-MS	EGCG interacts with proteins involved in metabolic pathways, DNA synthesis and pro-apoptotic signalling pathways	Sahadevan, Binoy, Vechalapu, Nanjan, & Sadhukhan, 2023

Note: *EGCG: epigallocatechin gallate, TP: tea polyphenols, GTP: green tea polyphenols, GTE: green tea extract, DBTVS: Dianhong black tea volatile substances, DE: gel electrophoresis, RPC: reverse phase chromatography, TSC: titan sphere chromatography, SELDI: surface-enhanced laser desorption/ionization, MALDI: matrix-assisted laser desorption/ionization, MS: mass spectrometry, TOF: time of flight, TOFu: time-of-flight upgrade, QTOF: quadrupole time-of-flight, ESI: electrospray ionization, TMT: tandem mass tag.

### 5.1 Anti-cancer effects

To better understand the anticancer effect of theophylline (from *C. sinensis*) in inhibiting growth of melanoma, an aggressive skin cancer, two patient-derived human melanoma initiating cell lines (with distinct pathways involved in melanoma development) (Fig. 2A) were exposed to theophylline (Table 1 P15). In melanoma initiating cells, proteins involved in cell proliferation and survival and related to the cellular membrane were abundant, while apoptosis pathways were less abundant. Theophylline-treated melanoma initiating cells mainly expressed proteins involved in dendrite formation and cytoskeleton dynamics, suggesting that theophylline may induce cellular differentiation. Combining proteomics with molecular analyses, theophylline was proven to affect cytoskeleton reorganization and induce cellular differentiation of melanoma initiating cells. These findings highlight the potential of theophylline supplementation as an adjunctive treatment for melanoma (Cordella et al., 2019).

To explore novel chemotherapeutic targets of EGCG for use in lung cancer treatment, its effects on human lung adenocarcinoma A549 cells (Fig. 2A) were studied (Table 1 P14). EGCG suppressed hepatoma-derived growth factor (HDGF), a protein known to promote A549 cellular proliferation, migration and differentiation. Further investigation into the biological consequences of HDGF inhibition revealed that EGCG enhanced chemotherapy-induced apoptosis, thereby increasing the sensitivity of A549 cells to chemotherapy. These findings suggest that targeting HDGF with EGCG could offer a promising strategy for the chemoprevention and treatment of lung cancer (Flores-Pérez et al., 2016).

The molecular mechanism of EGCG-triggered apoptosis of cancer cells was investigated in human urinary bladder carcinoma TSGH-8301 cells (Fig. 2A, Table 1 P11). Proteins, including HSP27, porin, tropomyosin 3 isoform 2, prohibitin, and keratin 5, 14 and 17, which are involved in cellular process such as apoptosis, mitochondrial function, cytoskeletal organization, and cellular integrity, were affected by EGCG in TSGH-8301 cells and led to activation of apoptotic pathways. Decreased HSP27 and p-AKT levels were specifically linked to the activation of apoptosis by EGCG. EGCG also had antitumour effects with a decrease in tumour size in BALB/c nu/nu athymic mice (a tumour-forming mouse model injected with TSGH-8301 cells). EGCG therefore had anticancer and antitumour effects on cancer cells in part through activation of apoptosis (Chen et al., 2011).

To identify the proteins which are regulated by EGCG, in-situ chemoproteomics was done using a biotin-tagged probe of EGCG to pull down EGCG-interacting proteins (Table 1 P24). Human cervical carcinoma (HeLa) cells (Fig. 2A) were treated with the EGCG probe, cells were lysed and the biotin-tagged EGCG probe together with EGCG-bound proteins were extracted. Pathway analysis of the 160 EGCG-interacting proteins indicated the regulation of metabolic pathways such as glycolysis, fructose-galactose metabolism, pentose phosphate pathway, pyruvate metabolism, tricarboxylic acid (TCA) cycle and DNA synthesis. EGCG also interacted with proteins involved in pro-apoptotic signalling pathways. Since many of these pathways are involved in carcinogenesis, identifying them as EGCG targets can help explain the biological activity of EGCG and ultimately allow for its optimal therapeutic use (Sahadevan, Binoy, Vechalapu, Nanjan, & Sadhukhan, 2023).

Green tea polyphenols (GTPs) exhibit antitumor activity through actin remodelling, increasing cell adhesion and reducing cell motility, which are key processes in the transition of premalignant to malignant cells. Researchers sought to identify molecular targets of green tea extract (GTE) to prevent cell motility and therefore inhibit lung cancer malignant development by using proteomics (Table 1 P9). GTE caused a dose dependent increase in lamin protein levels in human lung adenocarcinoma A549 cells (Fig. 2A). Lamins are filamentous proteins forming part of the nuclear membrane, and increased lamin levels increases cell stiffness thereby decreasing cell motility and therefore inhibiting metastasis. Since metastasis is a key process driving cancer cell spread and therefore cancer progression and poor patient outcomes, limiting cell motility represents an important therapeutic strategy. Lamins may therefore represent an interesting factor to investigate with regards to metastasis of lung cancers (Lu et al., 2009). Subsequent bioinformatic analyses also indicated an important role of the higher abundance of heterogeneous nuclear ribonucleoprotein A2/B1 (HNRNPA2B1) and C1/C2 (HNRNPC1C2) as well as poly(rC)-binding protein 1 (PCBP1) – proteins known to regulate the cell cytoskeleton – in the anticancer activity of GTE (Zamanian-Azodi, Rezaei-Tavirani, Esmaeili, & Rezaei Tavirani, 2021).

### 5.2 Vascular protective effects

The mechanism of cardioprotective effect of EGCG was investigated in a chronic heart failure model in rats (Fig. 2D, Table 1 P16). Proteomic analysis indicated that energy metabolism may be a key driver of the cardioprotective effects of EGCG since energy metabolism processes such as oxidative phosphorylation and lipid metabolism as well as respiratory chain components were positively affected by EGCG. Additional analyses indicated that EGCG could improve myocardial energy metabolism and cardiac function. EGCG may therefore function as possible treatment strategy for cardiac dysfunction by enhancing energy metabolism (Mou et al., 2022).

The mechanism of EGCG in preventing age-related vascular diseases was investigated using proteomics in early and later passages of human dermal microvascular endothelial cells (HDMEC) (Fig. 2A; Table 1 P8). EGCG induced the levels of moesin, rho guanosine-5′-diphosphate-dissociation inhibitor and actin proteins, all of which are related to cytoskeleton formation, compared to untreated aged cells. Since aging cells typically have declined cytoskeletal integrity, the induction of these factors likely helps increase the cytoskeletal integrity and function of aged cells, thereby protecting against age-related vascular diseases (Lee, Chung, Bang, & Lee, 2006).

### 5.3 Neuroprotective effects

To elucidate the molecular basis of the neuro-rescue ability of EGCG, proteomics coupled with transcriptomics were used to provide a comprehensive overview of pathways affected by EGCG in long-term serum deprived SH-SY5Y neuroblastoma cells, as a progressive neurotoxic model (Fig. 2A; Table 1 P10). EGCG’s iron chelation ability caused reduced levels of prolyl 4-hydroxylase, which would in turn increase the levels of hypoxia-inducible factor (HIF) thereby preventing oxidative neuronal death (Weinreb et al., 2007, Weinreb et al., 2008). EGCG was subsequently shown to significantly reduce cell death, which was confirmed by 3-(4,5-dimethylthiazol-2-yl)-2,5-diphenyltetrazolium bromide (MTT) assays (Weinreb, Amit, & Youdim, 2008). A higher abundance of cytoskeletal proteins and binding proteins which play a crucial role in developing of neurons was observed upon exposure to EGCG. EGCG also increased the levels of energy balance regulating proteins, and since cellular energy was associated with mitochondria, mitochondrial metabolism, which suggested that the neurorescue effect of EGCG may be associated with its antioxidant effect (Weinreb, Amit, & Youdim, 2008). Since oxidative damage and iron accumulation have been linked to neurodegenerative disease pathology, preventing oxidative stress while increasing iron chelation have become of interest for Alzheimer’s and Parkinson’s disease treatment (Weinreb, Amit, & Youdim, 2007).

GTEs and environmental enrichment (EE) improve cognition in down syndrome (DS), part of the mechanism lies in inhibiting dual specificity tyrosine-phosphorylation-regulated kinase 1A (Dyrk1A) protein phosphorylation activity (De Toma et al., 2020, De Toma et al., 2019). The mechanism of this improved cognition was explored using proteomics and phospho-proteomics in both Dyrk1A overexpressing and trisomy 21 DS mouse models (Fig. 2C; Table 1 P20). In Dyrk1A overexpressing DS model, proteins enriched in antioxidant activity were restored to wild-type levels by GTE, EE and their combination, with the combination restoring the most proteins. GTE, EE and the combination was also able to rescue the phosphoproteins enriched in synaptic neurogenesis-related pathways, with GTE showing the biggest rescue ability. The effect of GTE, EE and the combination was confirmed to be as a result of partial restoration of Dyrk1A expression level (De Toma, Ortega, Aloy, Sabidó, & Dierssen, 2019).

In the trisomy 21 mouse model (Table 1 P21), GTE, EE and the combination also partially restored some of the protein levels to wild-type levels, with the combination showing the biggest restoration effect. All treatments partially rescued the phosphoproteins, with GTE showing the biggest effect. The treatments were able to rescue some of the 45 deregulated kinases observed in the trisomy 21 model. The rescued proteins showed enrichment for synapse/neurodevelopment, chromatin/immune systems, microtubule/cytoskeleton, and GTPase activity. Since EGCG had previously been shown to inhibit histone deacetylation and have DNA methyl transferase activity, it was interesting that in this study, all treatments were also able to re-balance the epigenetic state, which was known to be compromised in DS. All treatments also caused a reduction in the levels of histone H3 acetylation thereby rescuing chromatin structures by making them less repressed and subsequently promoting the expression of memory promoting genes. The treatments were not only able to restore some of the effects of trisomy 21 on the proteome and phospho-proteome, but also affected a much broader range of proteins not directly associated with trisomy 21 (De Toma et al., 2020). All treatments were correlated with increased cognition assessed by behavioural analyses. The use of GTE and GTE in combination with EE to enhance cognition in intellectual disabilities such as DS was therefore suggested (De Toma et al., 2020, De Toma et al., 2019).

### 5.4 Effect on metabolic syndrome

The mechanism of the antihyperglycemic effect of green tea was investigated using a mouse model for diabetes (Fig. 2C; Table 1 P1). Both diabetes and green tea altered the serum protein profiles of the mice. The researchers were however surprised that green tea was unable to reverse the diabetes induced modifications. Only one protein peak specific to diabetes, a 4 211 Da protein, was found to be significantly decreased in the diabetic state and further decreased after green tea administration. They suggested a role for this protein in the antihyperglycemic effect of green tea, although its specific mechanism of action is not well understood, because the levels were further decreased compared to the diabetes control. Although unable to identify the specific protein at the time, it was speculated to be a haemoglobin related protein (Tsuneki et al., 2004). Interestingly, research has since shown that tea consumption can lead to a reduction in haemoglobin levels, thereby indicating that haemoglobin related proteins are affected by tea (Sachdev & Jothipriya, 2017), although the role of the 4 211 Da protein as a haemoglobin related protein remains speculative.

Non-alcoholic fatty liver disease (NAFLD) is a common chronic liver disease characterized by accumulation of fat droplets in liver and is associated with metabolic syndrome. The mechanism by which EGCG inhibits lipid buildup in liver was investigated in HepG2 liver cells that were experimentally induced to accumulate lipids in order to mimic the steatotic conditions of NAFLD (Fig. 2A; Table 1 P4). EGCG altered proteins linked to energy metabolism, lipid metabolism, glycometabolism and redox control. EGCG treatment also caused less abundance of proteins linked to oxidative metabolism and energy expenditure and increased the levels of those involved in lipid metabolism and glycometabolism. EGCG therefore suppresses hepatic gluconeogenesis, improves fat oxidation, and modifies redox balance. Target proteins involved in the process of EGCG-induced reduction of cellular lipid accumulation, as well as the molecular mechanisms underlying this effect were therefore identified (Liu et al., 2013).

The effect of Fuzhuan brick tea in a NAFLD rat model was also investigated (Fig. 2D; Table 1 P13). Fuzhuan brick tea was able to restore the levels of many proteins that were normally induced by a high fat diet to normal diet levels. It reduced lipogenesis and glycolysis while enhancing fatty acid oxidation through the activation of the TCA cycle and respiratory chain. Fat accumulation in rat livers was also significantly reduced, thereby indicating that Fuzhuan brick tea can serve as potential treatment strategy for NAFLD (Liu et al., 2015).

### 5.5 Intestinal health benefits

The intestines produce exosomes. These extracellular vesicles contain nucleic acids and proteins which play a role in cell-to-cell communication. The effect of EGCG on the protein content of exosomes was investigated in human intestinal epithelial cells derived from Caco-2 cells (Fig. 2A; Table 1 P18). EGCG decreased the number and size of exosomes while changing the protein content. Proteins involved in response to wound healing, regulation of bodily fluid levels, skin development and negative regulation of blood coagulation were more abundant in the EGCG treated cell exosomes. EGCG also decreased the galectin-3-binding protein (LGALS3BP), which plays a role in inflammation, while increasing fibronectin 1, a glycoprotein crucial for cell adhesion, wound healing, and tissue repair, indicating a potential mechanism for EGCG in supporting intestinal health (Yano, Suzuki, & Hara, 2023).

The anti-inflammatory property of GTP for treating colon inflammation was investigated in a Crohn’s disease (CD) mouse model (Fig. 2C; Table 1 P12). Analysis of the colon tissue proteome indicated 44 differentially expressed protein spots representing 33 unique proteins upon supplementation with GTP. These proteins were associated with cellular processes, including cellular stress, immune response and inflammation, as well as cellular assembly, organizational processes and signalling pathways. Integration of proteomics and transcriptomics data indicated that protein and gene expression of cellular detoxification process were upregulated and immune and inflammatory protein and gene expression downregulated by GTP. This was correlated with reduced tissue inflammation upon GTP supplementation in histological examinations. GTP also downregulated gene expression of pathways associated with the fibrogenesis process, which is involved in tissue injury response and generation of scar tissue. GTP could therefore potentially be helpful in treatment of inflammatory colon diseases, by reducing inflammation and preventing build-up of scar tissue in the colon (Barnett et al., 2013).

The mechanism of oolong tea in the regulation of circadian rhythm disorders, which are known to induce gut dysbiosis and cause similar effects to obesity, was investigated in C57BL/6J mice colonized with human stool (Fig. 2C; Table 1 P17). Mice were exposed to complete darkness (representing the circadian rhythm disturbed group) or complete darkness with dietary supplementation with oolong tea. Proteins were extracted from stool and a taxonomic proteomic analysis done. Oolong tea caused differential abundance in proteins involved in carbohydrate metabolism, global and overview maps, amino acid metabolism, genetic information processing and environmental information processing. This change in host metabolism pathways by oolong tea was correlated with increased *Bacteroides* and decreased *Faecalibacterium*, *Mitsuokella*, and *Ruminococcus* levels. Since intestinal microbiota regulated the host’s energy harvesting from food, increased levels of beneficial microbiota would be helpful to restore the host’s energy metabolism. Oolong tea therefore has the potential to help with treatment of circadian rhythm disorders by overcoming gut dysbiosis associated with the disease and having a prebiotic effect (Guo et al., 2019).

### 5.6 Antibacterial effects

The mechanism of the antibacterial effect of tea polyphenols on *Escherichia coli* was studied by proteomics (Fig. 2B; Table 1 P5). Proteins involved in cellular defence were more abundant while proteins involved in carbon and energy metabolism and amino acid biosynthesis were less abundant in the presence of tea polyphenols. Tea polyphenols therefore caused a concentration dependent death of *E. coli* by inhibiting metabolic processes and preventing protein synthesis (Cho, Schiller, Kahng, & Oh, 2007).

The role of cell membrane damage in the antibacterial effects of tea polyphenols on *Pseudomonas aeruginosa*, an opportunistic pathogen, was investigated (Fig. 2B; Table 1 P6). Tea polyphenols were found to reduce proteins involved in the TCA cycle, potentially disrupting this critical metabolic pathway. Additionally, proteins related to fatty acid biosynthesis were diminished, likely impairing the cell membrane’s structure, as fatty acids are integral components. Glycine metabolism, protein biosynthesis and DNA metabolism related proteins were also negatively impacted by tea polyphenols. Given glycine’s importance in the production of cell wall components and membrane-associated molecules, its dysregulation could hinder cell wall formation. These alterations in key proteins likely led to metabolic disruption and compromised membrane integrity, contributing to the antibacterial effects of tea polyphenols observed in *P. aeruginosa* (Yi, Zhu, Fu, & Li, 2010).

Tea polyphenols also caused less abundance of proteins involved in energy production, primary metabolism and lipopolysaccharide biosynthesis and dysregulated chaperone and protein biosynthesis proteins in another opportunistic pathogen, *Serratia marcescens* (Fig. 2B; Table 1 P7). Tea polyphenols therefore also inhibited *S. marcescens* growth by causing metabolic disorder and disruption of the cell membrane (Yi et al., 2014).

*Klebsiella pneumonia* is a bacterium associated with intra-abdominal sepsis and pneumonia and has become increasingly resistant to imipenem, a drug used for multidrug resistant gram-negative bacteria. To investigate whether EGCG could work synergistically with imipenem to inhibit growth of imipenem-resistant *K. pneumonia*, twelve clinical isolates were exposed to either imipenem or imipenem in combination with EGCG (Fig. 2B; Table 1 P2). Proteins involved in stress response and detoxification were significantly increased upon exposure to EGCG, whereas the proteins related to energy metabolism, protein synthesis and DNA metabolism were significantly decreased by EGCG. Concomitantly, while EGCG at half its minimum inhibitory concentration was not bactericidal, this concentration of EGCG significantly reduced the concentration of imipenem needed to kill imipenem-resistant *K. pneumonia*, thereby indicating a synergistic effect. The use of EGCG in combination with imipenem was therefore suggested for *K. pneumonia* infections (Cho, Oh, & Oh, 2011).

### 5.7 Other health benefits

The mechanism by which theacrine protects skin from UV-induced photodamage was investigated in female Institute of Cancer research (ICR) mice exposed to UV, followed by post-exposure to theacrine (Fig. 2C; Table 1 P23). Theacrine increased proteins involved in glycolysis/gluconeogenesis and tyrosine metabolism while decreasing proteins involved in DNA replication, cell cycle and apoptosis. The reduced expression of Bcl-2, Bcl-2-associated X protein (BAX) and Caspase-3 proteins was associated with decreased apoptosis in the presence of theacrine. Molecular docking analyses further suggested that theacrine can interact directly with BAX, Bcl-2, Caspase-3, and p53, supporting the idea that inhibition of apoptosis contributes to its protective effect against UV-induced skin damage (He et al., 2023).

Oxidative stress plays a role in bone loss, especially during oestrogen deficiency. Since GTP has antioxidant activity, its mechanism of protection of ovariectomized rats (oestrogen deficient) against bone loss was investigated (Fig. 2D; Table 1 P3). GTP increased the superoxide dismutase-1 (SOD-1) and adenosine triphosphate synthase (ATPase) levels thereby having antioxidant activity. GTP also lowered catechol-*O*-methyltransferase levels, which means catechol oestrogens that can exert oestrogenic effects would accumulate and therefore could help mitigate the negative effects of oestrogen loss on bone health. Both antioxidant and increased catechol oestrogens therefore contribute to GTP’s ability to promote bone health (Shao, Chen, Lu, Shen, & Gao, 2011).

The ability of Dianhong black tea volatile substances (DBTVS) to combat the negative effects of alcohol on liver injury was investigated in specific pathogen-free (SPF) male Sprague-Dawley (SD) rats (Fig. 2D; Table 1 P19). The combined proteomic and transcriptomic analyses indicated that DBTVS inhibited several lipid metabolism related proteins by influencing pathways involved in PPAR signalling, steroid biosynthesis, fatty acid metabolism and fatty acid degradation. This was correlated with improved lipid metabolism in the rats. DBTVS may also impact inflammatory signalling which could potentially reduce inflammation. DBTVS therefore effectively inhibited alcoholic liver injury by reducing lipid accumulation in the liver and modulating hepatic lipid metabolism (Gao et al., 2023).

## 6 Impact of advances in proteomics

Proteomic studies on the role of teas in human health have significantly advanced our understanding of their mechanisms of action. The depth of data obtained from these studies depends on the proteomic techniques employed (Table 1). Many studies used two-dimensional gel electrophoresis (2DE) to separate proteins by molecular weight and isoelectric point, followed by mass spectrometry (MS) to identify differentially abundant proteins. The integration of liquid chromatography offered an additional separation dimension, improved protein resolution, recovery, and identification, particularly for low-abundance or hydrophobic proteins. The 2DE method, however, provides limited data as only a few differentially abundant protein spots identified by staining are usually analysed by MS for identification of the proteins within the spots.

In addition to the development of protein separation techniques, comprehensive developments in MS technologies, which involves peptide ionization, ion separation and detection as well as mass over charge analysis, have also significantly increased the detection abilities of mass spectrometers (Arevalo et al., 2020, Li et al., 2021a, Noor et al., 2021). The shift to automated systems, advanced liquid chromatography coupled with advanced MS techniques, coupled with advanced bioinformatic analyses, led to the development of shotgun proteomics, which identifies thousands of proteins simultaneously (Oliveira et al., 2014, Zhan et al., 2018).

While shotgun proteomics generates comprehensive datasets, it requires sophisticated data analysis and advanced computational tools (Noor et al., 2021, Oliveira et al., 2014, Zhan et al., 2018). The protein quantification step in shotgun proteomics is for instance done by either label-free protein quantification, relying on advanced data processing coupled with careful normalization to compare protein abundances between samples, or protein labelling allowing for improved quantification accuracy and precision as well as multiplexing of samples leading to reduced batch effects (Bantscheff, Lemeer, Savitski, & Kuster, 2012). Most tea studies used label-free quantification, with only two employing TMT-labelling and one isotope labelling (Table 1).

Shotgun proteomics requires complex bioinformatic tools including pathway analysis which allows for the identifications of links among thousands of proteins identified as differentially abundant. By linking proteins to specific pathways, the global effect on the entire pathway can be assessed thereby offering a more holistic understanding of the tea’s effects. Bioinformatics tools together with protein databases have evolved, enhancing protein identification and pathway analysis, especially compared to earlier databases often not containing certain proteins and therefore making them impossible to identify (Tsuneki et al., 2004).

Advances in proteomic technologies, particularly shotgun proteomics, have expanded the scope of research on tea’s health benefits compared to traditional 2DE methods. However, combining modern MS with 2DE protein separation is gaining renewed interest due to its advantages in specialized applications such as isoform analysis and post-translational modifications (Marcus et al., 2020, Zhan et al., 2018). A deeper insight into tea’s mechanism will improve its therapeutic use in treating human diseases.

## 7 Role of metabolomic studies in determining health benefits of teas

**Fig. 3:**
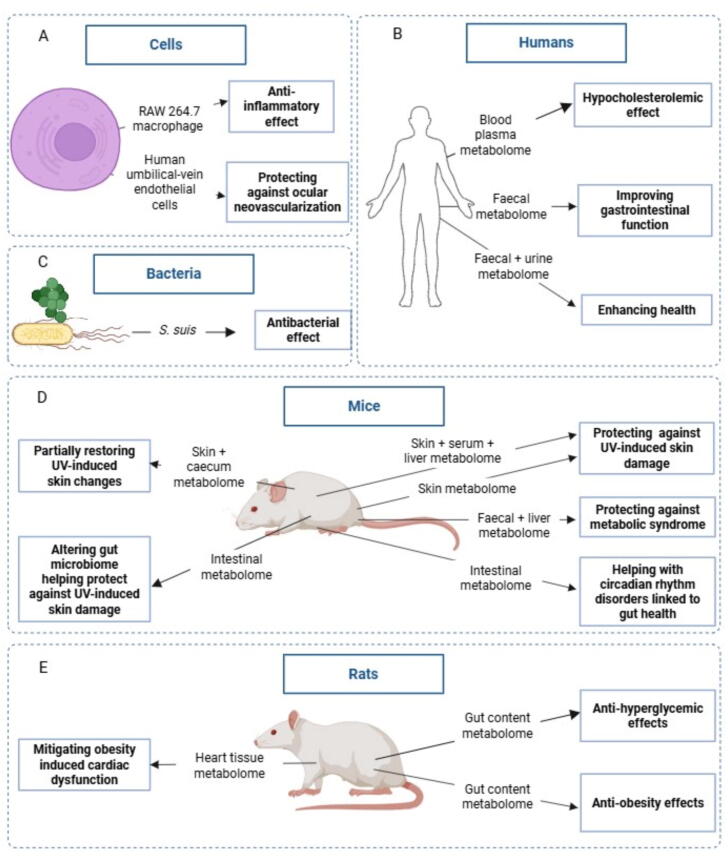
Health benefits of teas indicated by metabolomic analyses in various models including (A) cells, (B) humans, (C) bacteria, (D) mice and (E) rats. This image was created in Biorender.com.

A literature search identified 16 studies using metabolomics to investigate the health benefits of *C. sinensis* teas. It is important to note that studies focussing on determining the specific metabolic breakdown products of tea compounds during metabolism were not relevant to this review. No metabolomic studies investigating the health benefits of *A. linearis* and *Cyclopia* spp*.* teas were identified. The metabolomics strategies and MS techniques used in *C. sinensis* studies, organized in terms of strategy and year, are summarized in Table 2. The various models used for investigating the health benefits of *C. sinensis* teas by metabolomics are summarized in Fig. 3.

**Table 2:** Metabolomic studies aimed at understanding health benefits of *C. sinensis* teas.

	Teas/compounds	Study objectives	Models	Tissues	Metabolomics strategies*		Key findings	References
					Strategies	Techniques		
M1	GTE, EGCG, M1, M2	Ability to prevent ocular neovascularization	HUVEC cells	−	Untargeted	Micro-LC QTOF-ESI-MS	All four tea treatments induced cellular apoptosis, GTE was considered superior	Chu et al., 2018
M2	GTP	Antibacterial activity	*S. suis in vitro* cultures	−	Untargeted	UPLC-QTOF/MS	Green tea affected metabolites involved in numerous pathways leading to bacterial death	Gao et al., 2022b
M3	EGCG	Antibacterial activity	*S. suis in vitro* cultures	−	Untargeted	LC-Triple-TOF-ESI-MS	EGCG altered metabolites involved in ‘ABC transporters’, ‘glycolysis/gluconeogenesis’, and ‘aminoacyl-tRNA biosynthesis’ inducing bacterial death	Gao et al., 2022a
M4	Green tea	Ability to help with circadian rhythm disorders	SPF C57BL/6J male mice	Intestine	Untargeted	UPLC-Triple-TOF-MS	Green tea increased metabolic regulation, neuroprotection, decreased carcinogens and altered circadian rhythm metabolites, showing potential for treatment	Zhang et al., 2022
M5	Oolong tea	Effect on microbiota and gut health	Healthy adult humans	Faeces	Untargeted	UPLC-MS/MS	Oolong tea improved gastrointestinal function by enhancing health-beneficial bacteria	Li et al., 2023
M6	*L*-Theanine	Cardioprotective effect	Male SPF Wistar rats	Heart tissue	Untargeted	LC-MS/MS	*L*-theanine helped mitigate the increase in body weight and cardiac dysfunction caused by a high fat diet	Fang et al., 2024
M7	Green tea	Protection against UVB-induced skin damage	Female Skh:HR-1 hairless mice, chronic UVB model	Skin	Untargeted + Targeted	Untargeted: UPLC-QTOF-MS and GC-TOF-MS Targeted: LTQ XL-MS, (TriVersa NanoMate)	Green tea decreased ascorbic acid, amino acids, lysophospholipids and several organic acid levels while increasing fatty acids, lysoPCs, and carbohydrates and altering nucleobases, reversing the effects of UVB	Jung et al., 2015
M8	Green tea	Protection against UVB-induced skin damage	Female Skh:HR-1 hairless mice	Skin, serum and liver	Untargeted + Targeted	Untargeted: UPLC-QTOF-MS and GC-TOF-MS Targeted: LTQ XL-MS, (TriVersa NanoMate)	Green tea attenuated the effect of UVB on ascorbate, amino acid, steroid and nucleotide metabolism and the urea cycle in the skin	Jung et al., 2017a
M9	Green tea	Protection against UVB-induced skin damage	Female Skh:HR-1 hairless mice	Intestine	Untargeted + Targeted	Untargeted: UPLC-Q-TOF-MS and GC-TOF-MS Targeted: UPLC-ESI-QqQ-MS/MS	Green tea decreased most amino acids, fatty acids, lipids and bile acids while increasing the nucleobases and carbohydrates providing protection against UVB damage	Jung et al., 2017b
M10	GTP	Effects on metabolic dysfunction	Female SD rats	Gut content	Untargeted + Targeted	Untargeted: GC–MS Targeted: HPLC and GC–MS	Green tea reduced carbohydrates and bile constituents, modified amino acid metabolism and elevated vitamin production with anti-obesity effects	Zhou, Tang, Shen, & Wang, 2018
M11	GTP	Health benefits of tea	Healthy females	Faecal and urine	Untargeted + Targeted	UPLC-QTOF-ESI-MS/MS	GTP decreased the aromatic amino acid levels in urine but not in faeces	Zhou, Zhang, Arikawa, & Chen, 2019
M12	GTE, EGCG, catechin	Effect on metabolic dysfunction	Male C57BL/6J mice	Faecal and liver	Untargeted + Targeted	Untargeted: HILIC-UPLC–MS/MS Targeted: HPLC-ESI-MS	GTE, EGCG and catechin uniquely affected the metabolomes while protecting against metabolic dysfunction	Sun, Dey, Bruno, & Zhu, 2022
M13	GTE	Effects on metabolic dysfunction	Healthy, males	Blood plasma	Pseudo-targeted	HPLC-MS/MS and GC–MS (MRM-mode)	Green tea had a hypocholesterolaemia effect	Hodgson et al., 2014
M14	Green tea, EGCG, theanine, caffeine	Protective affect against UV-induced skin damage	Female Skh:HR-1 hairless mice	Skin and caecum	Pseudo-targeted	UPLC-QTOF-MS and GC-TOF-MS (MRM-mode)	GTE reversed the skin metabolomic alterations caused by UV	Jung et al., 2019
M15	GTP	Effect on metabolic dysfunction	Female SD rats	Gut content	Pseudo-targeted	HILIC-LC-MS/MS (SRM-mode)	GTP enhanced the efficiency of systematic energy conversion and reduced blood glucose, triglycerides and total cholesterol	Zhou, Tang, Shen, & Wang, 2020
M16	Se-enriched green tea	Ability of Se enrichment to enhance tea health benefits	RAW264.7 macro-phages	−	Pseudo-targeted	UHPLC/ESI-Q-Orbitrap	Both teas activated anti-inflammatory pathways, with Se enriched teas affecting more pathways	Fan, Jia, Du, & Shi, 2022

Note: *LC: liquid chromatography, QTOF: quadrupole time-of-flight, ESI: electrospray ionization, MS: mass spectrometry, UPLC: ultra performance liquid chromatography, TOF: time of flight, GC: gas chromatography, LTQ: linear ion trap quadrupole, QqQ: triple quadrupole mass spectrometer, HILIC: hydrophilic interaction liquid chromatography, MRM: multiple reaction monitoring, SRM: selected reaction monitoring, UHPLC: ultra high-performance liquid chromatography.

M1: a mixture with the major purified catechins found in GTE, M2: mixture of EGCG, GC, GCG, and ECG.

### 7.1 Anti-inflammatory properties

Selenium has anti-inflammatory, antioxidant and metal detoxification activities, which is suggested to enhance the health functions of tea polyphenols. Selenium-enriched green tea leaf extract has higher and distinct polyphenol content than regular green tea leaf extract. To investigate the ability of selenium enrichment to enhance the anti-inflammatory properties of green tea, RAW264.7 macrophages were exposed to the enriched and unenriched extract while induced with a chemical irritant inducing inflammation (Fig. 3A, Table 2 M16). The macrophage metabolome was extracted and pseudo-targeted metabolomic analysis was done. Several metabolic processes related to anti-inflammatory properties were affected by both selenium-enriched and regular tea polyphenol extracts, which included aminoacyl-tRNA biosynthesis valine, leucine and isoleucine biosynthesis valine, leucine and isoleucine degradation, starch and sucrose metabolism, histidine metabolism and purine metabolism. Selenium enriched tea polyphenols additionally altered phenylalanine, tyrosine and tryptophan biosynthesis, phenylalanine metabolism, and pantothenate and CoA biosynthesis while vitamin B6 metabolism and nicotinate and nicotinamide metabolism were metabolic pathways uniquely affected by regular tea polyphenols. Selenium enriched tea polyphenols mainly promoted the synthesis of antioxidants thereby preventing inflammation, while nicotinate and nicotinamide metabolism and vitamin B6 metabolism were specific metabolic pathways involved in the anti-inflammatory effect of regular tea polyphenols. Selenium enrichment therefore alters the anti-inflammatory properties of tea polyphenols, but further research is necessary to determine if one is superior than the other (Fan, Jia, Du, & Shi, 2022).

### 7.2 Effect on skin

To investigate the mechanism by which green tea protects skin against UV-induced damage, female Skh:HR-1 hairless mice were exposed to UVB in a chronic exposure model and their feed supplemented with green tea (Fig. 3D, Table 2 M7). Metabolomics of skin indicated that while stearoyl and docosahexaenoyl-lysophosphatidylcholines (two lysophospholipids), fatty acids and carbohydrates were decreased by exposure to UVB irradiation, green tea was able to attenuate these changes. While UVB increased the levels of ascorbic acid, amino acid, the remaining lysophospholipids, ceramides and several organic acids, green tea was able to decrease these levels. Dietary green tea supplementation therefore reduced the alterations in skin metabolome caused by chronic UVB exposure (Jung et al., 2015).

In a follow-up study, skin metabolomics data was integrated with skin microarray gene expression data to further explore metabolic pathways involved in the protective effects of green tea against UVB-induced skin damage (Fig. 3D, Table 2 M8). Skin metabolomics identified 34 skin associated metabolites and 49 ceramides showing discriminant levels between the normal diet without UVB, normal diet with UVB and green tea supplemented diet with UVB exposure groups. While UVB increased ornithine, ascorbic acid, ethanolamine, lysophosphatidylethanolamine and ceramide levels in the skin, green tea supplementation was able to attenuate these changes, thereby alleviating the effects of UVB on the skin metabolome. This was correlated with reduced UVB-induced skin damage observed in the presence of green tea supplementation. Integration of the skin metabolomics and skin microarray data indicated that green tea significantly attenuated the effect of UVB on ascorbate metabolism, amino acid metabolism, steroid metabolism, nucleotide metabolism, and the urea cycle. Since ascorbate metabolism and the urea cycle are essential in skin structure maintenance and barrier integrity, this provides a likely mechanism by which green tea is able to protect against UVB damage (Jung et al., 2017a). Since UV induced skin damage related molecules were thought to be able to reach the blood circulation and potentially affect the internal organs, serum and liver metabolomic analysis were also done. As with the skin metabolome, green tea supplementation was also able to attenuate most of the serum metabolite changes caused by UVB exposure. However, surprisingly, green tea further intensified the liver metabolite changes caused by UVB exposure, indicating that both UVB exposure and green tea may contribute to inflammation in the liver. Further investigation of these contradicting results is therefore necessary (Jung et al., 2017a).

To investigate whether the protective effects of green tea against UVB-induced skin damage were linked to the gut microbiome, the intestinal microbiome was analysed and correlated with the intestinal metabolome in this chronic UV exposure model (Fig. 3D, Table 2 M9). While UVB exposure caused predominant *Bacilli* and *Bifidobacteriales* levels, green tea enhanced the intestinal diversity increasing *Clostridia* and *Erysipelotrichia*, *Allobaculum* and *Lachnoclostridium* levels. Correspondingly, green tea altered the intestinal metabolome, increasing amino acids, fatty acids, lipids, and bile acids while decreasing nucleobases and carbohydrate levels. Changes in the microbiome caused by green tea may therefore be a contributing mechanism by which green tea is able to protect against UVB-induced skin damage (Jung et al., 2017b).

To further investigate the link between changes in the microbiome and the ability of green tea to protect against UVB-induced skin damage, researchers sought to combine skin and intestinal metabolomics with microbiome analysis. Instead of using a chronic UVB exposure model, Skh:HR-1 hairless mice were exposed to a single dose of UV after pre-exposure to GTE, EGCG, theanine or caffeine (Fig. 2D, Table 2 M14). UV without prior supplementation increased the levels of most amino acids, organic compounds, nucleobases, and lysophospholipids and decreased levels of carbohydrates and fatty acids in the skin. Short-term supplementation with GTE prior to UV exposure was able to reverse these skin metabolic patterns for several amino acids, organic compounds, and nucleobases, as well as most fatty acids and lysophospholipids. The individual components (EGCG, theanine and caffeine) were however only able to prevent some, but not all the skin metabolome changes caused by UV. Only a few intestinal metabolites were affected by UVB compared to skin metabolites, while there was big variation in the metabolite profiles when supplementation was done with the tea extract or different tea components. Correspondingly, green tea changed the microbiome with higher levels of pro-biotic bacteria, *Lactobacillus* species and *Bifidobacteria*. The change in microbiome and intestinal metabolome may therefore contribute to the change in the skin metabolome although the exact mechanistic link remains to be determined (Jung et al., 2019).

### 7.3 Effect on metabolic syndrome

To compare the effect of EGCG, catechin and GTE on the restoration of host metabolism to alleviated obesity, male C57BL/6J mice were fed a high fat diet or high-fat diet supplemented with EGCG, catechin or GTE (Fig. 3D, Table 2 M12). When comparing the faecal (microbiome) metabolic pathways affected by GTE and catechin, there were nine differentially altered pathways including TCA cycle and tyrosine metabolism. Only three metabolic pathways were however differently affected between GTE and EGCG group. Indicating a more similar liver metabolome alteration between EGCG and GTE compared to catechin. When comparing liver metabolome, glycolysis and gluconeogenesis pathways were significantly different between GTE and EGCG groups, while only hepatic tyrosine metabolism was different between catechin and GTE supplemented groups. This indicates that the purified catechins from green tea have a unique contribution towards the liver metabolome and may therefore play distinct roles in protecting against metabolic dysfunction which could lead to obesity (Sun, Dey, Bruno, & Zhu, 2022).

The impact of *L*-theanine in modulating obesity-induced cardiac dysfunction was investigated in specific-pathogen-free (SPF) Wistar rats and cardiac tissues and blood samples collected for metabolomic and transcriptomic analysis (Fig. 3E, Table 2 M6). *L*-theanine significantly altered the metabolite expression in the rats with obesity. The differentially abundant metabolites were linked to 55 pathways, including degradation of aromatic compounds, drug metabolism-cytochrome P450, renin-angiotensin system, phenylalanine metabolism, and tyrosine metabolism. The metabolomic and transcriptional profiles were correlated in terms of 18 overlapping KEGG pathways identified, 10 of which were related to metabolism. *L*-theanine may therefore help mitigate the increase in body weight and cardiac dysfunction caused by obesity (Fang et al., 2024).

In a placebo-controlled intervention study, healthy physically active male participants, consumed a diet supplemented with GTE (Fig. 3B, Table 2 M13). Blood was collected after fasting and plasma was isolated before metabolomic profiling. Dietary supplementation with GTEs significantly reduced hippuric acid and cholesteryl esters while increasing caffeine, salicylic acid, taurine, dihydroxyphenylethylene glycol, serotonin, certain triglycerides, sphingosines and fatty acids levels. Green tea therefore lowered cholesterol thereby improving human health and suggesting potential benefits for those with metabolic syndrome (Hodgson et al., 2014).

Long-term GTP supplementation was shown to change the intestinal microbiome, enhance *Bacteroidetes* and *Oscillospira* families and almost deplete the *Peptostreptococcaceae* family in rats. Metabolomic profiling was subsequently done to analyse the intestinal content of rats receiving GTP to better understand the gut-microbiota dependent metabolic changes induced by GTP (Fig. 3E, Table 2 M10). The gut content was analysed by long-term dietary supplementation with various concentrations of GTP. Both time of supplementation and concentration of GTP affected the metabolomic patterns in rats. Pathway analysis and global metabolite-gene network analysis identified a reduction in calorific carbohydrates, modification in amino acid metabolism, elevation in vitamin production and decrease in bile constituents in rats receiving GTPs. These changes can be linked with anti-obesity effect of GTP (Zhou, Tang, Shen, & Wang, 2018).

The metabolomics analysis of aforementioned study was however geared towards the more hydrophobic metabolites because reverse phase chromatography was used for separation. Since the major components of gut-microbiota metabolites are more hydrophilic than hydrophobic components, a hydrophilic interaction liquid chromatography (HILIC) silica column was subsequently used to analyse more hydrophilic metabolites in the same rat model (Fig. 3E, Table 2 M15). Enhanced cysteine and methionine metabolism, glycine, serine and threonine metabolism, valine, leucine and isoleucine biosynthesis and purine metabolism were observed in the six months GTP supplementation group. GTP consumption significantly enhanced the TCA and urea cycle metabolites, thereby enhancing the efficiency of systematic energy conversion. A reduction in blood glucose, triglycerides and total cholesterol was also observed. The metabolomics data correlated with microbiome changes caused by GTP enhancing advantageous microbes. The effect of GTP on these pathways and the microbiome therefore helps explain the anti-hyperglycaemic effect of GTP (Zhou, Tang, Shen, & Wang, 2020).

### 7.4 Antibacterial effects

The effect of GTP on *Streptococcus suis* was investigated using metabolomics (Fig. 3C, Table 2 M2). GTP inhibited growth, damaged cells and decreased the virulence of *S. suis*. This phenotype was correlated with the effect of GTP on metabolites involved in ABC transporters, pyrimidine metabolism, protein digestion and absorption, alanine, aspartate and glutamate metabolism, aminoacyl-tRNA biosynthesis, carbon fixation in photosynthetic organisms, arginine biosynthesis and fatty acid biosynthesis providing potential pathways involved in the antibacterial effect of GTP (Gao et al., 2022b).

The effect of EGCG on *S. suis* growth, haemolytic activity, biofilm formation, and pathogenicity was also investigated using metabolomics combined with proteomic analysis (Fig. 2, Fig. 3; Table 1 P22, Table 2 M3). EGCG caused 121 differentially abundant metabolites, 75 of which were higher and 46 lower abundances. Pathways including protein digestion and absorption, ABC transporters, aminoacyl-tRNA biosynthesis, mineral absorption, pyrimidine metabolism, alanine, aspartate, and glutamate metabolism, glycerophospholipid metabolism, arginine biosynthesis and fatty acid biosynthesis were altered by EGCG. The proteomics analysis indicated that EGCG caused differentially abundant proteins linked to cell development and division, components of cell walls and membranes, drug resistance, environmental adaptation, and haemolytic activity. Integration of the metabolomics and proteomic data indicated 70 shared KEGG pathways important in the antibacterial effect of EGCG on *S. suis*, with the top three being ABC transporters, glycolysis/gluconeogenesis, and aminoacyl-tRNA biosynthesis (Gao et al., 2022a). Green tea and EGCG may therefore be further explored as treatment for *S. suis* infections.

### 7.5 Other health benefits

Ocular neovascularization, the abnormal growth of capillaries in the eyes, leads to vision decline and ultimately blindness. Human umbilical-vein endothelial (HUVEC) cells were exposed to GTE, EGCG, a mixture of the major purified catechins (M1) or a mixture of four catechins known to be dominant in the intraocular compartment (EGCG, GC, GCG, and ECG) (M2) in a neovascularization cell model (Fig. 3A, Table 2 M1). Untargeted metabolomics analysis revealed that GTE affected purine, pyrimidine, phenylalanine, vitamin B6, cysteine, methionine, pantothenate, CoA and glycophospholipid metabolism, leading to suppression of biosynthesis of cellular building blocks and oxidative phosphorylation while promoting synthesis of membrane lipids and growth factors. EGCG altered vitamin B6, valine and leucine, tyrosine, and glycerophospholipid metabolism. M1 altered glycosylphosphatidylinositol, riboflavin, and pantothenate and CoA metabolism pathways while M2 altered pantothenate and CoA metabolism. Different tea components therefore caused distinct metabolomics profiles, although all four tea treatments induced cellular apoptosis. GTE was suggested as superior to the individual components for potential treatment of ocular neovascularization (Chu et al., 2018).

The benefits of oolong tea on gut microbiota and metabolism were investigated in 28 healthy adults divided into two groups, one’s diet supplemented with oolong tea and another abstaining from tea and coffee. Faecal samples were collected for gut microbial and metabolomic investigations (Fig. 3B; Table 2 M5). Metabolomic analysis identified 23 differentially abundant metabolites, among which 14 were up- and nine down-regulated in the oolong tea consuming group. These metabolites include carboxylic acids and derivatives, benzene and substituted derivatives, fatty acyls, steroids and steroid derivatives. The identified metabolites were found to be associated with 10 metabolic pathways including neomycin, kanamycin, and gentamicin biosynthesis, tyrosine metabolism, alpha-linolenic acid metabolism, neuroactive ligand-receptor interaction, glycine, serine and threonine metabolism, arachidonic acid metabolism, folate biosynthesis, protein digestion and absorption, and drug metabolism cytochrome P450. The group that consumed oolong tea had enhanced growth of health-beneficial bacteria and inhibition of harmful bacteria accompanied by an improvement in gastrointestinal function (Li et al., 2023).

To investigate the health benefits of green tea, healthy postmenopausal females consumed dietary GTP supplementation or placebo for twelve months, whereafter metabolomic analyses of faecal and urine samples were done (Fig. 3B, Table 2 M11). GTP decreased the aromatic amino acid levels, including indoxyl sulphate, phenylacetylglutamine, and hippuric acid, in urine but not faeces. Since indoxyl sulphate and other aromatic metabolites have previously been associated inflammation, which could lead to cardiovascular and kidney disease, the ability of GTP to reduce their levels likely improves human health. Interestingly, the faecal microbiome was not altered by long-term GTP supplementation in this study. The alteration of aromatic amino acid levels may therefore contribute to the health benefit of green tea consumption in humans (Zhou, Zhang, Arikawa, & Chen, 2019).

A complex relationship between intestinal microbiome and the host’s circadian clock has been identified, which leads to pathophysiological changes if out of balance. Researchers sought to determine if green tea supplementation, which is known to affect the microbiome, could help with circadian rhythm disorders. SPF C57BL/6 J male mice were either exposed to cycles of light (control group), complete darkness (causing circadian rhythm disorders), or complete darkness while being fed green tea (Fig. 3D, Table 2 M4). Intestinal metabolites were analysed and correlated to the intestinal bacterial content, while single-cell RNA-sequencing was done on hypothalamus cells to investigate the levels of circadian rhythm related genes. supplementation with green tea significantly increased the levels of metabolites, including *O*-acetyl-*L*-carnitine, *trans*-caffeic acid, and daidzein, which are involved in metabolic regulation and neuroprotection. Concomitantly, the levels of carcinogens involved in damage of brain’s nervous system were significantly reduced in the green tea group. Green tea also altered metabolites and metabolic pathways such as alanine, aspartate and glutamate metabolism, arginine biosynthesis and C5-branched dibasic acid metabolism, which were involved in circadian rhythm. The abnormal abundance of intestinal flora because of circadian rhythm disorder could be overcame by supplementation with green tea. A strong correlation between the metabolome and microbiota was also established. Single-cell RNA-sequencing indicated that green tea increased the number of astrocytes and oligodendrocytes in the hypothalamus and significantly increased the expression of genes involved in circadian rhythm, *Csnk1d*, *Clock*, *Per3*, *BhIhe41*, and *Cry2*, compared to the circadian rhythm disorder group. This suggests that green tea improves circadian rhythm disorders and suggests pathways involved in this protective effect which can be further explored (Zhang et al., 2022).

## 8 Impact of advances in metabolomics

The studies using metabolomics to investigate the role of tea in human health clearly made meaningful contributions to determine the mechanism of action of tea and tea components. The number of metabolites identified by mass spectrometry based on metabolomics are dependent on the metabolomics strategy, metabolite extraction techniques, metabolite separation techniques, MS instrumentation and methodologies, and bioinformatic databases available for metabolite identification (Chen et al., 2022, Ren et al., 2018, Ribbenstedt et al., 2018, Zeki et al., 2020). The metabolomics strategies with a brief description of the separation techniques and MS strategies followed in tea studies are summarized in Table 2.

The following metabolomics approaches can be followed: targeted, untargeted, or a combination of both targeted and untargeted. Targeted metabolomics identifies and quantifies a predicted set of metabolites using standard quantification, and is therefore hypothesis driven leading to a limited scope. Untargeted metabolomics is an exploratory technique, which provides a more comprehensive overview of as many as possible metabolites to discover novel pathways affected. Targeted metabolomics therefore offers accurate quantification, but a limited scope. Whereas, untargeted metabolomics provides comprehensive datasets, but runs the risk of not quantifying as accurately or being unable to identify novel metabolites (Ribbenstedt, Ziarrusta, & Benskin, 2018).

The combination of both targeted and untargeted metabolomics is the most advantageous. Combining targeted and untargeted metabolomics can entail many strategies including running both techniques separately followed by integration of generated data. Alternatively, untargeted metabolomics can be ran followed by targeted metabolomics on the same system using specialized machine settings, which has been referred to as pseudo-targeted metabolomics. While these techniques have been confusingly termed and used interchangeably, there are difference which should be considered as thoroughly summarized (Amer, Deshpande, & Bird, 2023). While a few tea studies used untargeted metabolomics, most used a combination of both untargeted and targeted or pseudo-targeted approaches (Table 2).

Combining both untargeted and targeted metabolomics approaches, with optimal metabolite extraction and separation, modern MS instrumentation and methodologies, and complex bioinformatic analyses will lead to optimal metabolomics analyses. This has great potential for the identification of novel pathways and integration with known pathways affected by tea, which will help elucidate the mechanism of action of tea and tea components.

## 9 Discussion and perspectives

The use of tea as an alternative or complementary treatment for various diseases is of special interest, due to its well documented health benefits. Before implementation of the use of tea in treatment can be done, it is necessary to identify the mechanisms of action of tea and its bioactive compounds. Understanding the mechanism of action will ultimately allow for pharmacological integration with various disease treatment plans, achieving the maximum benefit while minimizing the risk of potential contraindications with other medications.

Proteomics and metabolomics allow for simultaneously studying the changes to numerous proteins and metabolites, which are caused by teas. Analysing the metabolic pathways to which these proteins and metabolites belong, allows for assembling a picture of the biological activity and mechanism of action of the tea. While many reviews have explored the role of proteomics or metabolomics in determining the health benefits of various natural remedies (Hasanpour et al., 2020, Khan et al., 2022, Lao et al., 2014, Oyenihi et al., 2021, Shi et al., 2016), this review is the first time to summarize the role of these techniques in determining the health benefits of teas and tea components.

The literature review identified studies using proteomics and metabolomics for investigating the health benefits of *C. sinensis* teas and tea components. Proteomics has been used to investigate the anti-cancer, vascular protective, neuroprotective, skin protective, bone health support, liver disease preventative, metabolic syndrome preventative, intestinal health, prebiotic and antibacterial health properties. Metabolomics studies indicated the anti-inflammatory, vascular protective, skin protective, prevention of metabolic syndrome, prebiotic and antibacterial properties of *C. sinensis* teas.

Surprisingly, no studies investigating the role of proteomics and metabolomics in the health benefits for *A. linearis* and *Cyclopia* spp. teas were identified, which is a major limitation of the current study review. Since proteomics and metabolomics studies clearly significantly contributed to investigating the mechanism of action of *C. sinensis* teas, the use of these techniques to investigate the health benefits of *A. linearis* and *Cyclopia* spp. tea plants would also be beneficial. This is especially important since the three tea plants have been shown to contain unique bioactive compounds, which likely diversifies their mechanisms in affecting human health.

While each study using proteomics and metabolomics to investigate the health benefits of *C. sinensis* tea has clearly contributed significantly towards better understanding the mechanisms of action of tea, it is important to notice certain limitations of these studies and research gaps which are evident from this review. Firstly, it was noted that the exact bioactive compounds of tea used in many of studies were not determined. Since the composition of each tea varies depending on various factors including demographics, preparation method and fermentation levels, determining the exact bioactive composition and establishing and standardizing proper dosages are imperative before it could be incorporated into any official treatment regimens. Secondly, these studies have been done in numerous models ranging from single cell to animal and human model (Fig. 2, Fig. 3). In each model, the bioavailability of the tea bioactive compounds would differ, which could affect their biological properties. Enhancing the bioavailability of tea bioactive compounds has been an important focus in tea research, since many compounds have been shown to have poor bioavailability (Chaudhary et al., 2023). The studies in mouse, rat and human models indicate that, as expected, proteomic and metabolomic changes induced by tea are organ specific, making studying the effect of tea on diseases with multi-organ involvement challenging. These factors would be essential to consider before ultimately incorporating tea into ‘official treatment regimens’.

Lastly, proteomics studies indicating the health benefits of *C. sinensis* tea were published between 2004 and 2024 (Table 1) and metabolomics studies between 2014 and 2024 (Table 2). Considering the significant advancements in the sample preparation methods, protein/peptide separation methods, MS technology, data analysis and databases within this time range (previously discussed), the limitation of each study based on these factors needs to be considered. For instance, while the identification of proteins was initially impossible (Tsuneki et al., 2004) (Table 1 P1), the identification of entire metabolic pathways affected by tea is now possible.

With the development of proteomics and metabolomics technologies, there was a clear increase in the amount of data, and therefore also the number of pathways identified as associated with the health benefits of *C. sinensis* tea. Furthermore, integration of proteomics and metabolomics analysis enabled corroboration of data leading to an enhanced understanding of the pathways involved (Table 1 P22/Table 2 M3). Integration of transcriptomic with proteomics (Table 1 P10, P12, P19) or metabolomics data (Table 2 M4, M6, M8) also allowed for data confirmation and corroboration in some of these studies, even though it is important to note that transcriptional changes are not always reflected at the protein or metabolite level, or conversely. Integrating multiple omics will lead to the generation of complex profiles spreading over multiple biological levels (Babu & Snyder, 2023) and therefore provide an even more encompassing picture of biological effect of tea on human health.

Proteomics and metabolomics offer significant potential in human health and medicine, while the integration of multi-omics will empower future precision health approaches. There are however several limitations and challenges to the use of these techniques in precision health, which are elegantly explored in these reviews (Al-Amrani et al., 2021, Babu and Snyder, 2023, Zhang et al., 2020). Some considerations include: (1) Protein expression varies with environment and cell type, indicating the importance of ultimately exploring the proteomic profiles in humans to apply findings to human health. (2) While protein levels may be indicated as more or less abundant, this does not necessarily speak to protein activities because of post-translational modifications. This highlights the importance of complementing standard proteomics with approaches that specifically analyse protein modifications, such as phosphoproteomics, because protein abundance alone does not necessarily reflect activity. Post-translational modifications like phosphorylation can critically regulate protein function, and assessing these modifications provides more accurate insights into the pathways that are actually active or affected (Table 1 P20, P21). (3) Sample preparation methods and MS run protocols greatly affect proteins which are purified and assessed, making the establishment of comparable proteomics methods essential (yet extremely challenging) to allow for better inter-study comparisons (Al-Amrani, Al-Jabri, Al-Zaabi, Alshekaili, & Al-Khabori, 2021). (4) While sample type, sample preparation and MS run protocols also affect the metabolome analysed, one of the biggest challenges in metabolomics is the lack of identification of a substantial portion of the human metabolome. This hinders comprehensive metabolomic phenotyping (Zhang, Li, Xu, & Dou, 2020). (5) Multi-omics remains especially challenging, since it involves combining diverse omics datasets requiring advanced computational tools to ensure meaningful data integration (Babu & Snyder, 2023). (6) Proteomics, metabolomics, and multi-omics all require extensive sample preparation, expensive machinery and have complex computational demands. (7) Furthermore, it is important to consider how variations in study design make comparing studies challenging (Al-Amrani et al., 2021, Babu and Snyder, 2023, Zhang et al., 2020).

Despite their limitations, each study using proteomics and metabolomics to investigate the health benefits of *C. sinensis* tea provides valuable information towards understanding the mechanisms of tea and tea components. With the popularity of *A. linearis* and *Cyclopia* spp. teas increasing worldwide due to their unique properties, it is imperative that studies using proteomics and metabolomics to investigate health benefits are also done for these teas. Each study serves as a stepping stone towards better understanding the mechanisms of tea in advancing human health. Despite not being included in official treatment plans, tea will continue to be widely consumed for its health benefits, as people have long relied on it as a natural remedy. Understanding these health benefits by making use of proteomics, metabolomics and multi-omics will ultimately allow for optimal beneficial application of these well-loved natural resources to human health.

## CRediT authorship contribution statement

**Danicke Willemse:** Conceptualization, Methodology, Investigation, Validation, Data curation, Formal analysis, Visualization, Writing – original draft, Writing – review & editing. **Mariam Rado:** Conceptualization, Methodology, Investigation, Validation, Data curation, Formal analysis, Visualization, Writing – original draft, Writing – review & editing. **Mariska Lilly:** Validation, Writing – review & editing, Supervision.

## Declaration of competing interest

The authors declare that they have no known competing financial interests or personal relationships that could have appeared to influence the work reported in this paper.

## References

[b0005] Abdul N.S., Marnewick J.L. (2023). What has been the focus of rooibos health research: A bibliometric overview. Journal of Herbal Medicine.

[b0010] Abdullah A.T.M., Sayka M.I., Rahman M.M., Sharif M., Khan T.A., Jahan S. (2024). Tea (*Camellia sinensis*) cultivated in three agro-ecological regions of Bangladesh: Unveiling the variability of methylxanthine, bioactive phenolic compound, and antioxidant activity. Heliyon.

[b0015] Afrifa D., Engelbrecht L., Eijnde B.O., Terblanche E. (2023). The health benefits of rooibos tea in humans (*Aspalathus linearis*)-a scoping review. Journal of Public Health in Africa.

[b0020] Agapouda, A., Butterweck, V., Hamburger, M., de Beer, D., Joubert, E., & Eckert, A. (2020). Honeybush extracts (*Cyclopia* spp.) rescue mitochondrial functions and bioenergetics against oxidative injury. *Oxidative Medicine and Cellular Longevity*, *2020*, 1948602. 10.1155/2020/1948602 PMC7428828 32831989

[b0025] Al-Amrani S., Al-Jabri Z., Al-Zaabi A., Alshekaili J., Al-Khabori M. (2021). Proteomics: Concepts and applications in human medicine. World Journal of Biological Chemistry.

[b0030] Aloo O.S., Kim D.G., Vijayalakshmi S., Aloo D.O., Ochola C.O., Oh D.H. (2024). Polyphenol constituents and impacts of fermented teas (*Camellia sinensis*) in human wellness. Food Bioscience.

[b0035] Amer B., Deshpande R.R., Bird S.S. (2023). Simultaneous quantitation and discovery (SQUAD) analysis: Combining the best of targeted and untargeted mass spectrometry-based metabolomics. Metabolites.

[b0040] Arevalo R., Ni Z.Q., Danell R.M. (2020). Mass spectrometry and planetary exploration: A brief review and future projection. Journal of Mass Spectrometry.

[b0045] Babu M., Snyder M. (2023). Multi-omics profiling for health. Molecular & Cellular Proteomics.

[b0050] Bantscheff M., Lemeer S., Savitski M.M., Kuster B. (2012). Quantitative mass spectrometry in proteomics: Critical review update from 2007 to the present. Analytical and Bioanalytical Chemistry.

[b0055] Barnett M.P.G., Cooney J.M., Dommels Y.E.M., Nones K., Brewster D.T., Park Z. (2013). Modulation of colonic inflammation in Mdr1a^−/−^ mice by green tea polyphenols and their effects on the colon transcriptome and proteome. The Journal of Nutritional Biochemistry.

[b0060] Chaudhary P., Mitra D., Das Mohapatra P.K., Oana Docea A., Myo E.M., Janmeda P. (2023). *Camellia sinensis*: Insights on its molecular mechanisms of action towards nutraceutical, anticancer potential and other therapeutic applications. Arabian Journal of Chemistry.

[b0065] Chaudhary S.K., Sandasi M., Makolo F., van Heerden F.R., Viljoen A.M. (2021). Aspalathin: A rare dietary dihydrochalcone from *Aspalathus linearis* (rooibos tea). Phytochemistry Reviews.

[b0070] Chen N.G., Lu C.C., Lin Y.H., Shen W.C., Lai C.H., Ho Y.J. (2011). Proteomic approaches to study epigallocatechin gallate-provoked apoptosis of TSGH-8301 human urinary bladder carcinoma cells: Roles of AKT and heat shock protein 27-modulated intrinsic apoptotic pathways. Oncology Reports.

[b0075] Chen Y., Li E.M., Xu L.Y. (2022). Guide to metabolomics analysis: A bioinformatics workflow. Metabolites.

[b0080] Cho Y.S., Schiller N.L., Kahng H.Y., Oh K.H. (2007). Cellular responses and proteomic analysis of *Escherichia coli* exposed to green tea polyphenols. Current Microbiology.

[b0085] Cho Y.S., Oh J.J., Oh K.H. (2011). Synergistic anti-bacterial and proteomic effects of epigallocatechin gallate on clinical isolates of imipenem-resistant *Klebsiella pneumoniae*. Phytomedicine.

[b0090] Chu K.O., Chan K.P., Chan S.O., Ng T.K., Jhanji V., Wang C.C. (2018). Metabolomics of green-tea catechins on vascular-endothelial-growth-factor-stimulated human-endothelial-cell survival. Journal of Agricultural and Food Chemistry.

[b0095] Cordella M., Tabolacci C., Senatore C., Rossi S., Mueller S., Lintas C. (2019). Theophylline induces differentiation and modulates cytoskeleton dynamics and cytokines secretion in human melanoma-initiating cells. Life Sciences.

[b0100] Dai W.D., Xie D.C., Lu M.L., Li P.L., Lv H.P., Yang C. (2017). Characterization of white tea metabolome: Comparison against green and black tea by a nontargeted metabolomics approach. Food Research International.

[b0105] Das C., Banerjee A., Saha M., Chatterjee S. (2022). A review of the health benefits of tea: Implications of the biochemical properties of the bioactive constituents. Current Research in Nutrition and Food Science Journal.

[b0110] De Beer D., Tobin J., Walczak B., Van Der Rijst M., Joubert E. (2019). Phenolic composition of rooibos changes during simulated fermentation: Effect of endogenous enzymes and fermentation temperature on reaction kinetics. Food Research International.

[b0115] De Toma I., Ortega M., Aloy P., Sabidó E., Dierssen M. (2019). DYRK1A overexpression alters cognition and neural-related proteomic pathways in the hippocampus that are rescued by green tea extract and/or environmental enrichment. Frontiers in Molecular Neuroscience.

[b0120] De Toma I., Ortega M., Catuara-Solarz S., Sierra C., Sabidó E., Dierssen M. (2020). Re-establishment of the epigenetic state and rescue of kinome deregulation in Ts65Dn mice upon treatment with green tea extract and environmental enrichment. Scientific Reports.

[b0125] El Oirdi M. (2024). Harnessing the power of polyphenols: A new frontier in disease prevention and therapy. Pharmaceuticals.

[b0130] Fan Z.B., Jia W., Du A., Shi L. (2022). Pseudo-targeted metabolomics analysis of the therapeutic effect of phenolics-rich extract from Se-enriched green tea (*Camellia sinensis*) on LPS-stimulated murine macrophage (RAW_264.7_). Food Research International.

[b0135] Fang C.Y., Shen Y.F., Wang F.Y., Zhang J.Y., Liu C., Luo F. (2024). Combined transcriptomics and metabolomics to elucidate the underlying beneficial mechanisms of L-Theanine in mitigating obesity-induced cardiac injury in rats. Journal of Functional Foods.

[b0140] Fang R., Redfern S.P., Kirkup D., Porter E.A., Kite G.C., Terry L.A. (2017). Variation of theanine, phenolic, and methylxanthine compounds in 21 cultivars of *Camellia sinensis* harvested in different seasons. Food Chemistry.

[b0145] Flores-Pérez A., Marchat L.A., Sánchez L.L., Romero-Zamora D., Arechaga-Ocampo E., Ramírez-Torres N. (2016). Differential proteomic analysis reveals that EGCG inhibits HDGF and activates apoptosis to increase the sensitivity of non-small cells lung cancer to chemotherapy. Proteomics Clinical Applications.

[b0150] Gao T.H., Fu J.J., Liu L., Bai J., Lv Y.J., Zhu Y.J. (2023). Transcriptome and proteomics conjoint analysis reveal anti-alcoholic liver injury effect of Dianhong Black Tea volatile substances. Food Science & Nutrition.

[b0155] Gao T., Ye F., Tan Y., Peng M., Yuan F., Liu Z. (2022). Metabolomics and proteomics analyses revealed mechanistic insights on the antimicrobial activity of epigallocatechin gallate against *Streptococcus suis*. Frontiers in Cellular and Infection Microbiology.

[b0160] Gao T., Ye F., Yuan F.Y., Liu Z.W., Liu W., Zhou D.N. (2022). Green tea polyphenols inhibit growth, pathogenicity and metabolomics profiles of *Streptococcus suis*. Microbial Pathogenesis.

[b0165] Guo T.T., Song D., Ho C.T., Zhang X., Zhang C.D., Cao J.X. (2019). Omics analyses of gut microbiota in a circadian rhythm disorder mouse model fed with oolong tea polyphenols. Journal of Agricultural and Food Chemistry.

[b0170] Hasanpour M., Iranshahy M., Iranshahi M. (2020). The application of metabolomics in investigating anti-diabetic activity of medicinal plants. Biomedicine & Pharmacotherapy.

[b0175] He Y., Zheng X.K., Hu Y.F., Deng L.H., Xu J., Wu S. (2023). Proteomics analysis to investigate the potential mechanism of theacrine against UV-induced skin photodamage. Photodermatology, Photoimmunology & Photomedicine.

[b0180] Hodgson A.B., Randell R.K., Mahabir-Jagessar-T K., Lotito S., Mulder T., Mela D.J. (2014). Acute effects of green tea extract intake on exogenous and endogenous metabolites in human plasma. Journal of Agricultural and Food Chemistry.

[b0185] Joubert E., de Beer D., Malherbe C.J., Muller M., Louw A., Gelderblom W.C.A. (2019). Formal honeybush tea industry reaches 20-year milestone–progress of product research targeting phenolic composition, quality and bioactivity. South African Journal of Botany.

[b0190] Joubert E., Fouche G., Vermaak I., Mulaudzi N., Chen W. (2023). *The South African Herbal Pharmacopoeia*.

[b0195] Joubert E., Schulz H. (2006). Production and quality aspects of rooibos tea and related products. a review. Journal of Applied Botany and Food Quality.

[b0200] Jung E.S., Park H.M., Hyun S.M., Shon J.C., Lakshmanan M., Noh M. (2017). Integrative metabolomic analysis reveals diet supplementation with green tea alleviates UVB-damaged mouse skin correlated with ascorbate metabolism and urea cycle. Metabolomics.

[b0205] Jung E.S., Park H.M., Hyun S.M., Shon J.C., Singh D., Liu K.H. (2017). The green tea modulates large intestinal microbiome and exo/endogenous metabolome altered through chronic UVB-exposure. PLoS One1.

[b0210] Jung E.S., Park H.M., Lee K.E., Shin J.H., Mun S., Kim J.K. (2015). A metabolomics approach shows that catechin-enriched green tea attenuates ultraviolet B-induced skin metabolite alterations in mice. Metabolomics.

[b0215] Jung E.S., Park J.I., Park H., Holzapfel W., Hwang J.S., Lee C.H. (2019). Seven-day green tea supplementation revamps gut microbiome and *Caecum*/skin metabolome in mice from stress. Scientific Reports.

[b0220] Karsen P.A., Lötze E., Valentine A.J., Hoffman E.W. (2022). Propagation and cultivation practices of honeybush (*Cyclopia* spp.) for the sustainable production of an export quality indigenous south african tea. Crop Science.

[b0225] Keet L., Magcwebeba T., Abel S., Louw A., Gelderblom W., Lilly M. (2024). Modulation of UVB-induced oxidative stress and inflammation in skin keratinocytes (HaCaT) utilising unfermented rooibos and honeybush aqueous extracts. Journal of Photochemistry and Photobiology.

[b0230] Khan F.B., Singh P., Jamous Y.F., Ali S.A., Abdullah U., S., (2022). Multifaceted pharmacological potentials of curcumin, genistein, and tanshinone IIA through proteomic approaches: An in-depth review. Cancers.

[b0235] Kim Y., Lee K.G., Kim M.K. (2016). Volatile and non-volatile compounds in green tea affected in harvesting time and their correlation to consumer preference. Journal of Food Science and Technology.

[b0240] Koláčková T., Kolofiková K., Sytařová I., Snopek L., Sumczynski D., Orsavová J. (2020). Matcha tea: Analysis of nutritional composition, phenolics and antioxidant activity. Plant Foods for Human Nutrition.

[b0245] Kumari R., Kumar S. (2024). Unlocking the therapeutic potential of *Camellia sinensis*: A comprehensive review of its pharmacological effects. Journal of Clinical Pharmacology and Therapeutics.

[b0250] Lao Y.Z., Wang X.Y., Xu N.H., Zhang H.M., Xu H.X. (2014). Application of proteomics to determine the mechanism of action of traditional chinese medicine remedies. Journal of Ethnopharmacology.

[b0255] Lee J.H., Chung K.Y., Bang D., Lee K.H. (2006). Searching for aging-related proteins in human dermal microvascular endothelial cells treated with anti-aging agents. Proteomics.

[b0260] Li A., Kou R.X., Liu H.W., Chen M.S., Wang J., Liu Q. (2023). Multi-omics analyses reveal relationships among polyphenol-rich oolong tea consumption, gut microbiota, and metabolic profile: A pilot study. Food Chemistry.

[b0265] Li C., Chu S.Y., Tan S.Y., Yin X.C., Jiang Y., Dai X.H. (2021). Towards higher sensitivity of mass spectrometry: A perspective from the mass analyzers. Frontiers in Chemistry.

[b0270] Li C.F., Ma J.Q., Huang D.J., Ma C.L., Jin J.Q., Yao M.Z. (2018). Comprehensive dissection of metabolic changes in albino and green tea cultivars. Journal of Agricultural and Food Chemistry.

[b0275] Li M.W., Shen Y., Ling T.J., Ho C.T., Li D.X., Guo H.M. (2021). Analysis of differentiated chemical components between zijuan purple tea and Yunkang green tea by UHPLC-orbitrap-MS/MS combined with chemometrics. Foods.

[b0280] Liu Z.H., Li Q., Huang J.N., Liang Q.L., Yan Y.J., Lin H.Y. (2013). Proteomic analysis of the inhibitory effect of epigallocatechin gallate on lipid accumulation in human HepG2 cells. Proteome Science.

[b0285] Liu Z.H., Lin Y., Zhang S., Wang D., Liang Q.L., Luo G.A. (2015). Comparative proteomic analysis using 2DE-LC-MS/MS reveals the mechanism of Fuzhuan brick tea extract against hepatic fat accumulation in rats with nonalcoholic fatty liver disease. Electrophoresis.

[b0290] Lu Q.Y., Yang Y.N., Jin Y.S., Zhang Z.F., Heber D., Li F.P. (2009). Effects of green tea extract on lung cancer A549 cells: Proteomic identification of proteins associated with cell migration. Proteomics.

[b0295] Maarman G.J., Lecour S. (2022). The potential benefit of rooibos (*Aspalathus linearis*) in pulmonary arterial hypertension: A short review. South African Journal of Botany.

[b0300] Malongane F., McGaw L.J., Nyoni H., Mudau F.N. (2018). Metabolic profiling of four South african herbal teas using high resolution liquid chromatography-mass spectrometry and nuclear magnetic resonance. Food Chemistry.

[b0305] Mandal D.S.K., Seth D.P. (2023). Active components of tea (*Camellia sinensis*) extracts and their beneficial application on human health. Sustainability, Agri, Food and Environmental Research.

[b0310] Marcus K., Lelong C., Rabilloud T. (2020). What room for two-dimensional gel-based proteomics in a shotgun proteomics world?. Proteomes.

[b0315] Masike K. (2021).

[b0320] Mou Q.H., Jia Z.L., Luo M., Liu L.J., Huang X.P., Quan J.J. (2022). Epigallocatechin-3-gallate exerts cardioprotective effects related to energy metabolism in pressure overload-induced cardiac dysfunction. Archives of Biochemistry and Biophysics.

[b0325] Noor Z., Ahn S.B., Baker M.S., Ranganathan S., Mohamedali A. (2021). Mass spectrometry-based protein identification in proteomics-a review. Briefings in Bioinformatics.

[b0330] Oliveira B.M., Coorssen J.R., Martins-de-Souza D. (2014). 2DE: The *Phoenix* of proteomics. Journal of Proteomics.

[b0335] Oyenihi O.R., Oyenihi A.B., Erhabor J.O., Matsabisa M.G., Oguntibeju O.O. (2021). Unravelling the anticancer mechanisms of traditional herbal medicines with metabolomics. Molecules.

[b0340] Pretorius L., Smith C. (2024). Green rooibos (*Aspalathus linearis*) promotes gut health: Insight into mechanisms. Journal of Ethnopharmacology.

[b0345] Pringle N.A., Koekemoer T.C., Holzer A., Young C., Venables L., van de Venter M. (2018). Potential therapeutic benefits of green and fermented rooibos (*Aspalathus linearis*) in dermal wound healing. Planta Medica.

[b0350] Reddy S., Rashed K., Marnewick J.L., Rautenbach F.G., Koekemoer T., van de Venter M. (2023). *Cyclopia intermedia* E. Mey protects against ROS-induced liver injury in HepG2/C3A cells. South African Journal of Botany.

[b0355] Ren J.L., Zhang A.H., Kong L., Wang X.J. (2018). Advances in mass spectrometry-based metabolomics for investigation of metabolites. RSC Advances.

[b0360] Ribbenstedt A., Ziarrusta H., Benskin J.P. (2018). Development, characterization and comparisons of targeted and non-targeted metabolomics methods. PLoS One1.

[b0365] Sachdev N.A., Jothipriya M. (2017). Effect of green tea on haemoglobin. IOSR Journal of Dental and Medical Sciences.

[b0370] Sahadevan R., Binoy A., Vechalapu S.K., Nanjan P., Sadhukhan S. (2023). *In situ* global proteomics profiling of EGCG targets using a cell-permeable and Click-able bioorthogonal probe. International Journal of Biological Macromolecules.

[b0375] Shao C.X., Chen L.X., Lu C.W., Shen C.L., Gao W.M. (2011). A gel-based proteomic analysis of the effects of green tea polyphenols on ovariectomized rats. Nutrition.

[b0380] Shi J., Cao B., Wang X.W., Aa J.Y., Duan J.N., Zhu X.X. (2016). Metabolomics and its application to the evaluation of the efficacy and toxicity of traditional Chinese herb medicines. Journal of Chromatography B.

[b0385] Stander M.A., Van Wyk B.E., Taylor M.J.C., Long H.S. (2017). Analysis of phenolic compounds in rooibos tea (*Aspalathus linearis*) with a comparison of flavonoid-based compounds in natural populations of plants from different regions. Journal of Agricultural and Food Chemistry.

[b0390] Sun X.W., Dey P., Bruno R.S., Zhu J.J. (2022). EGCG and catechin relative to green tea extract differentially modulate the gut microbial metabolome and liver metabolome to prevent obesity in mice fed a high-fat diet. The Journal of Nutritional Biochemistry.

[b0395] Tan J.F., Dai W.D., Lu M.L., Lv H.P., Guo L., Zhang Y. (2016). Study of the dynamic changes in the non-volatile chemical constituents of black tea during fermentation processing by a non-targeted metabolomics approach. Food Research International.

[b0400] Teixeira A.M., Sousa C. (2021). A review on the biological activity of *Camellia* species. Molecules.

[b0405] Tobin J. (2018).

[b0410] Tsuneki H., Ishizuka M., Terasawa M., Wu J.B., Sasaoka T., Kimura I. (2004). Effect of green tea on blood glucose levels and serum proteomic patterns in diabetic (db/db) mice and on glucose metabolism in healthy humans. BMC Pharmacology.

[b0415] Turkmen N., Sarı F., Velioglu Y.S. (2009). Factors affecting polyphenol content and composition of fresh and processed tea leaves. Akademik Gıda.

[b0420] Van Wyk B.E., Gorelik B. (2017). The history and ethnobotany of Cape herbal teas. South African Journal of Botany.

[b0425] Weinreb O., Amit T., Youdim M.B.H. (2007). A novel approach of proteomics and transcriptomics to study the mechanism of action of the antioxidant-iron chelator green tea polyphenol (-)-epigallocatechin-3-gallate. Free Radical Biology & Medicine.

[b0430] Weinreb O., Amit T., Youdim M.B.H. (2008). The application of proteomics for studying the neurorescue activity of the polyphenol (-)-epigallocatechin-3-gallate. Archives of Biochemistry and Biophysics.

[b0435] Windvogel S. (2019). Rooibos (*Aspalathus linearis*) and honeybush (*Cyclopia* spp.): From bush teas to potential therapy for cardiovascular disease. Nutraceuticals-Past, Present and Future. IntechOpen..

[b0440] Wong M., Sirisena S., Ng K. (2022). Phytochemical profile of differently processed tea: A review. Journal of Food Science.

[b0445] Xu J., Hu F.L., Wang W., Wan X.C., Bao G.H. (2015). Investigation on biochemical compositional changes during the microbial fermentation process of Fu brick tea by LC–MS based metabolomics. Food Chemistry.

[b0450] Xu J.Y., Wang M., Zhao J.P., Wang Y.H., Tang Q., Khan I.A. (2018). Yellow tea (*Camellia sinensis* L.), a promising chinese tea: Processing, chemical constituents and health benefits. Food Research International.

[b0455] Yano S., Suzuki K., Hara T. (2023). Proteomic profiling of intestinal epithelial-like cell-derived exosomes regulated by epigallocatechin gallate. BioFactors.

[b0460] Yi S.M., Wang W., Bai F.L., Zhu J.L., Li J.R., Li X.P. (2014). Antimicrobial effect and membrane-active mechanism of tea polyphenols against *Serratia marcescens*. World Journal of Microbiology and Biotechnology.

[b0465] Yi S.M., Zhu J.L., Fu L.L., Li J.R. (2010). Tea polyphenols inhibit *Pseudomonas aeruginosa* through damage to the cell membrane. International Journal of Food Microbiology.

[b0470] Zamanian-Azodi M., Rezaei-Tavirani M., Esmaeili S., Rezaei Tavirani M. (2021). Bioinformatics identification of green tea anticancer properties: A network-based approach. Research Journal of Pharmacognosy.

[b0475] Zeki Ö.C., Eylem C.C., Reçber T., Kır S., Nemutlu E. (2020). Integration of GC-MS and LC-MS for untargeted metabolomics profiling. Journal of Pharmaceutical and Biomedical Analysis.

[b0480] Zhan X., Li N., Zhan X., Qian S. (2018). Revival of 2DE-LC/MS in proteomics and its potential for large-scale study of human proteoforms. Med One.

[b0485] Zhang X.W., Li Q.H., Xu Z.D., Dou J.J. (2020). Mass spectrometry-based metabolomics in health and medical science: A systematic review. RSC Advances.

[b0490] Zhang Y.T., Cheng L., Liu Y.N., Zhang R.L., Wu Z.F., Cheng K.J. (2022). Omics analyses of intestinal microbiota and hypothalamus clock genes in circadian disturbance model mice fed with green tea polyphenols. Journal of Agricultural and Food Chemistry.

[b0495] Zhao F., Chen M.J., Jin S., Wang S.Y., Yue W.J., Zhang L.X. (2022). Macro-composition quantification combined with metabolomics analysis uncovered key dynamic chemical changes of aging white tea. Food Chemistry.

[b0500] Zhou J., Tang L.L., Shen C.L., Wang J.S. (2018). Green tea polyphenols modify gut-microbiota dependent metabolisms of energy, bile constituents and micronutrients in female Sprague-Dawley rats. The Journal of Nutritional Biochemistry.

[b0505] Zhou J., Tang L.L., Shen C.L., Wang J.S. (2020). Green tea polyphenols boost gut-microbiota-dependent mitochondrial TCA and urea cycles in Sprague-Dawley rats. The Journal of Nutritional Biochemistry.

[b0510] Zhou Y.Y., Zhang N.N., Arikawa A.Y., Chen C. (2019). Inhibitory effects of green tea polyphenols on microbial metabolism of aromatic amino acids in humans revealed by metabolomic analysis. Metabolites.

